# Dicationic Ion‐Pair Doping Enables Stable and High‐Performing Conducting Polymers for Organic Thermoelectrics

**DOI:** 10.1002/advs.77036

**Published:** 2026-08-15

**Authors:** Juhyung Park, Shubhradip Guchait, Tanmay Sinha, Cynthia Verduyckt, Vijitha Ignatious, Tzu‐Yi Yu, Heyi Xia, Altynay Kaidarova, Michael Ng, Siebe Detavernier, Martin Rosenthal, Maristella Alessio, Daniel Escudero, Guy Koeckelberghs, Francisco Molina‐Lopez

**Affiliations:** ^1^ Department of Materials Engineering KU Leuven Leuven Belgium; ^2^ Laboratory For Polymer Synthesis, Department of Chemistry KU Leuven Leuven Belgium; ^3^ Department of Chemistry KU Leuven Leuven Belgium; ^4^ Quantum Chemistry and Physical Chemistry, Department of Chemistry KU Leuven Leuven Belgium

**Keywords:** charge transport, dicationic, molecular doping, organic thermoelectrics, polymers, stability, TAB–2TFSI

## Abstract

Molecular doping is a key strategy for tuning the thermoelectric (TE) properties of conjugated polymers. Yet achieving both high TE performance and long‐term stability remains challenging, as counterions redistribute and microstructures reorganize under thermal and ambient exposure. Here we investigate TAB–2TFSI (TAB^2+^·2TFSI^−^), a dicationic ion‐pair dopant that couples a strong oxidizing TAB motif with two weakly coordinating TFSI^−^ counterions. Using poly(3‐hexylthiophene‐2,5‐diyl) (P3HT) as a model polymer, we benchmark TAB–2TFSI against state‐of‐the‐art p‐doping systems and find that it delivers the highest average electrical conductivity of 290 ± 30 S cm^−^^1^ and power factor of 40 ± 6 µW m^−^^1^ K^−^^2^, placing P3HT among the top high‐performing isotropic doped polymer thermoelectrics ever reported. Temperature‐dependent transport analysis, together with GIWAXS and AFM‐IR mapping of TFSI‐associated vibrational signatures, indicates morphology‐induced improved transport connectivity and a more homogeneous ionic landscape than for other dopants. Consistently, TAB–2TFSI exhibits the highest stability under inert aging and accelerated thermal/oxidative stress among all the doping systems tested, evidenced by minimal spectral and structural evolution over time and the most stable TE performance. Finally, we demonstrate the wide applicability of TAB–2TFSI as a dopant across multiple popular conjugated polymers, highlighting its material‐agnostic potential to boost simultaneously doping efficiency and stability.

## Graphical abstract

TAB–2TFSI enables efficient ion‐pair doping across a wide range of conjugated polymers, where the strongly oxidizing TAB core drives charge transfer, and the TFSI counterions promote favorable ionic compensation. This dicationic dopant promotes uniform dopant distribution, improves structural order, and increases carrier delocalization, establishing a general doping strategy leading to high thermoelectric performance and enhanced stability.

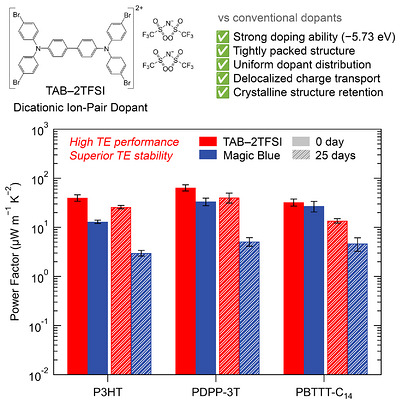

## 1 Introduction

Tuning the electronic properties of organic semiconductors is essential for a wide range of optoelectronic and energy‐harvesting devices [1, 2, 3, 4]. Molecular doping—based on redox‐driven charge transfer between dopant molecules and host material—has become a central approach for semiconducting polymers, enabling large enhancements in electrical conductivity (*σ*) and providing a versatile platform to interrogate structure–property relationships in doped semicrystalline films [5, 6, 7, 8, 9, 10]. However, the doped state is often dynamic rather than static: under thermal and ambient exposure, doped polymer films can undergo doping changes via gradual evolution in counterion distribution and relaxation of their semicrystalline packing [11]. As charge transport is tightly coupled to the ionic and microstructural landscape, such evolution can lead to substantial drift in *σ* and hence device performance over time [12, 13, 14, 15, 16].

This challenge is particularly pronounced in organic thermoelectrics (OTE), where continuous operation under a temperature gradient imposes prolonged thermal and ambient stress on the active layer [17, 18]. OTE performance is governed by the figure of merit *zT* = *α* ^2^ *σT*/*κ* and the power factor (*PF* = *α* ^2^ *σ*), where *α*, *T*, and *κ* are the Seebeck coefficient, absolute temperature, and thermal conductivity, respectively [19, 20, 21, 22, 23, 24, 25]. *α* is known to decrease with carrier concentration *n*, whereas according to the Drude model for metals (applicable to highly‐doped conjugated polymers), *σ* increases proportionally with *n* as *σ* = *qµn* (with *q* being the elementary charge). Therefore, *PF* peaks at a specific *n* and depends on carrier mobility *µ*. This makes maintaining an invariant doped state and morphology critical: even modest redistribution of ions or slow microstructural reorganization can shift *n* and *µ* away from their optimum, thereby eroding *PF* [26]. Accordingly, OTE‐relevant doping strategies must provide a broad tuning window in *n* for *PF* optimization while preserving high *µ* by minimizing carrier–ion coupling and respecting molecular order, and must be robust against time‐dependent evolution of both the ionic landscape and the doped microstructure [27].

To fulfill these stringent requirements, various p‐doping strategies have been explored. These approaches can be broadly classified into three main mechanisms based on how they manage charge transfer and counterion compensation:

1. Charge‐transfer doping [5]. In this conventional strategy, the single‐molecule neutral oxidant directly oxidizes the polymer, and the dopant‐derived reduced species remains in the film as the compensating counterion. However, typical single‐component oxidants —such as the molecular acceptor 2,3,5,6‐tetrafluoro‐7,7,8,8‐tetracyanoquinodimethane (F_4_TCNQ), or metal‐salt oxidants such as Iron(III) chloride (FeCl_3_), in which the redox‐active Fe(III) species drives oxidation— struggle to simultaneously satisfy the conditions required to achieve performing OTEs. This limitation fundamentally stems from the dual role these oxidizing species play (oxidant and counterion): upon accepting an electron from the polymer via charge transfer, the reduced dopant necessarily remains in the film as the compensating counterion [28, 29, 30]. In such a system, the thermodynamic driving force for doping (redox strength) and the physicochemical properties of the counterion are intrinsically linked. Consequently, achieving higher *n* requires employing stronger oxidants, but these typically yield reduced species that remain redox‐active or chemically unstable. These counterions are prone to reacting with ambient agents or causing off‐target reactions, which severely compromise the chemical stability of the doped material [31]. Furthermore, this single‐component approach leaves no degree of freedom to independently optimize the counterion electrostatic environment, as the counterion is strictly dictated by the choice of a strong oxidant. Thus, it is difficult to mitigate the carrier‐counterion interactions and associated microstructural disruption that may compromise charge transport without sacrificing the necessary redox drive. This inherent trade‐off makes it profoundly difficult to simultaneously optimize doping efficiency, *µ*, and stability [31].
2. Ion‐exchange doping [32]. A single‐molecule oxidant is added along with a salt. Like in charge‐transfer doping, the neutral oxidant oxidizes the polymer, becoming an anion. However, contrary to charge‐transfer doping, the reduced dopant does not remain as the polymer counterion. Instead, it exchanges its position with the anion from the added salt. Ion‐exchange can overcome the trade‐off between redox driving force and stability by decoupling the initial charge transfer step from the final counterion compensation. This decoupled approach offers two primary advantages. First, replacing a redox‐active dopant‐derived counterion with a closed‐shell, redox‐inactive spectator ion (supplied by an added salt) can significantly enhance the chemical stability of the doped state. Second, it enables the selection of a microstructure‐compatible counterion that does not perturb the polymer packing yet is sufficiently bulky to weaken carrier–counterion interactions, thereby preserving *µ*. However, the thermodynamics of exchange can be system‐dependent; for example, ion exchange with TFSI^−^ anions has been reported to be nonspontaneous in poly(3‐hexylthiophene) (P3HT), potentially limiting doping efficiency [33]. Moreover, anion‐exchange doping typically requires two components—an oxidant and a salt—so the final *n* and counterion environment can be sensitive to stoichiometry and processing sequence, complicating quantitative *PF* optimization.
3. Ion‐radical pair doping [34]. The dopant is a salt composed of a radical cation and a counteranion. The radical cation induces the oxidation of the polymer and becomes neutral in the process. Therefore, it yields its position to the anion from the salt, which, after intercalation, takes the role of the polymer counterion (similar to ion‐exchange doping but involving one anion replacing one neutral molecule instead of two anions exchanging positions). Examples of ion‐radical pair salts are tris(4‐bromophenyl)ammoniumyl bis(trifluoromethylsulfonyl)imide (TBPA–TFSI) or TBPA–SbCl_6_ (Magic Blue). In these systems, the radical TBPA^•+^ drives an initial one‐electron transfer from the polymer, while the accompanying anion serves as the counterion. Because ion–radical pair salts deliver both the oxidizing species and the counterion in a single component, they eliminate a separate anion‐exchange step and its associated kinetic/thermodynamic constraints. This single‐component nature can also make *PF* optimization more straightforward than in two‐component anion‐exchange protocols. Nevertheless, long‐term stability can still be limited by the intrinsic reactivity of radical cations and their potential to undergo side reactions in the solid state [34, 35].

Beyond counterion management, redox stoichiometry provides another way to tune transport and stability. In principle, double‐doping can enable a target carrier density at lower dopant loading, potentially reducing ionic/volumetric perturbations to semicrystalline morphology and improving operational stability [36]. To date, however, efficient double‐doping has been confined to exceptionally low‐ionization‐energy polymers (e.g., the glycolated polythiophene P(g2T‐TT), ionization energy (IE) ≈ 4.6 eV), consistent with the energetic requirement that the polymer IE must be low enough to enable electron transfer not only to the neutral dopant (electron affinity, EA^0^) but also to its anion (EA^−^) [26, 36]. As a result, double‐doping remains sparsely demonstrated for benchmark semicrystalline polymers such as poly(3‐hexylthiophene) (P3HT) [37] and poly(2,5‐bis(3‐alkylthiophen‐2‐yl)thieno[3,2‐b]thiophene) (PBTTT), and remains unreported for higher‐ionization‐energy backbones, including DPP‐based polymers such as a diketopyrrolopyrrole–terthiophene copolymer (PDPP‐3T).

Taken together, these considerations motivate finding dopant platforms that simultaneously (i) offer the potential for efficient double‐doping, (ii) establish a weakly coordinating, moderately large counterion environment that promotes charge delocalization, and (iii) remain chemically and kinetically stable in the solid state under thermal and ambient stress. The first two considerations aim at minimizing the volumetric disruption to the molecular packing by the counterion, ensuring high mobility, while the last consideration grants doping stability. Here, we investigate TAB–2TFSI (TAB^2^^+^·2TFSI^−^), a dicationic tetraaryl benzidine dopant first reported by Kurosawa et al. [35]. This doping system is fundamentally different from the three previously described categories. TAB–2TFSI is a dicationic ion‐pair salt, a compound comprising one TAB^2^^+^ diradical dication and two identical TFSI^−^ anions. Unlike the single‐anion ion‐radical pair salts, its dicationic core can, in principle, accept two electrons while providing two TFSI^−^ counterions per dopant, thereby coupling strong dication‐enabled oxidative double doping with a moderately large, weakly coordinating TFSI^−^ counterion environment. Using P3HT as a model semicrystalline polymer and benchmarking against representative doping strategies (i.e., charge‐transfer doping, anion‐exchange, and ion‐radical dopants), we aim to establish simultaneous improvements in peak thermoelectric (TE) performance (*σ* and *PF*) and stability under ambient and thermal stress. To connect performance and stability to the underlying morphological and electronic film state, we carry out extensive characterization over time, which includes: TE measurements, temperature‐dependent transport analysis, UV–vis‐NIR to assess the doping state, AFM‐IR spatial mapping of counterions, and grazing‐incidence wide‐angle X‐ray scattering (GIWAXS) analyses of polymer packing. Finally, we demonstrate the general applicability of TAB–2TFSI by unlocking top TE performance and enhanced stability in additional popular conjugated polymers.

## 2 Results and Discussion

### 2.1 Proposed Doping Scheme of TAB–2TFSI and Comparison with Benchmark Dopants

**FIGURE 1:**
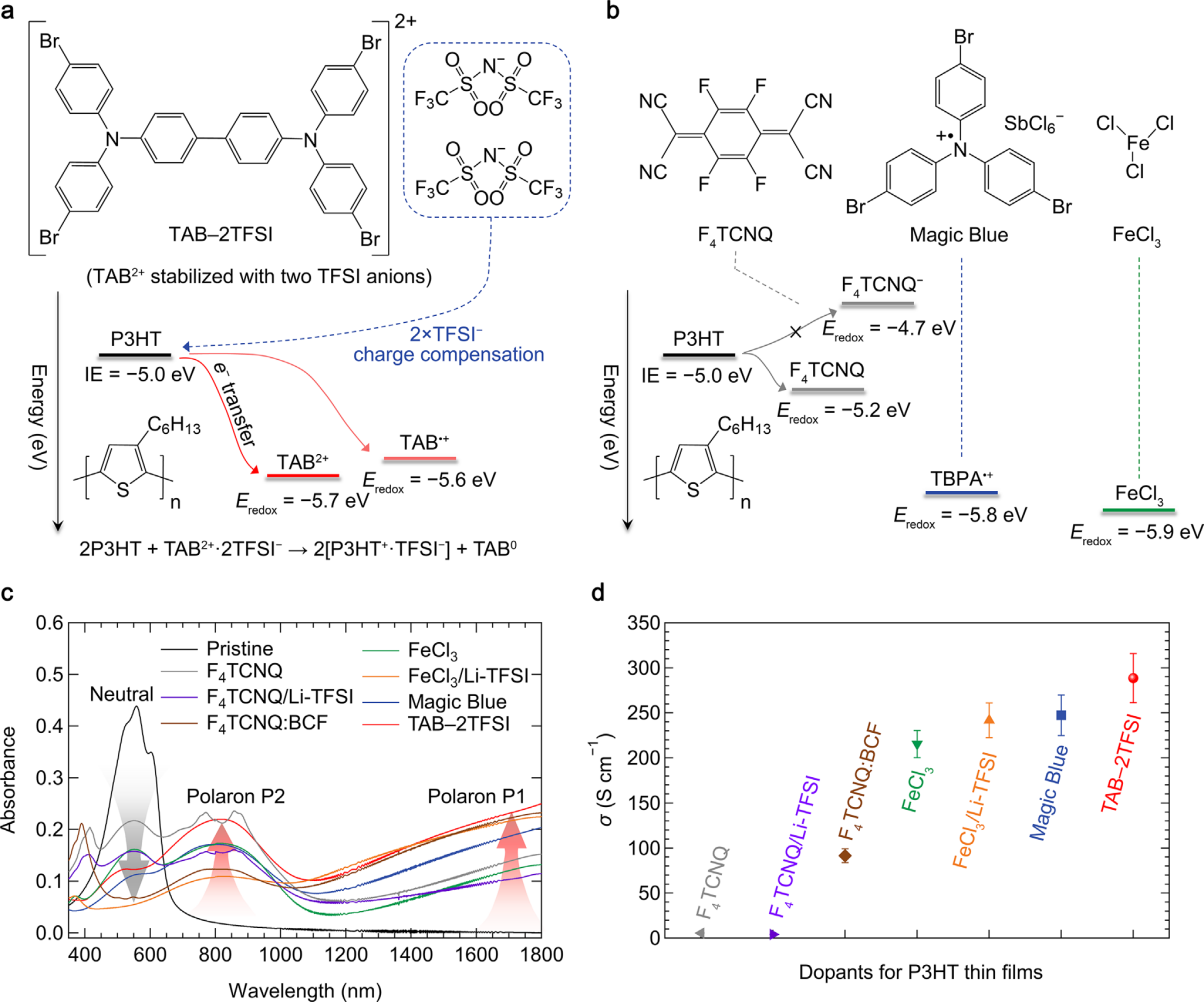
Chemical Structures, Doping Mechanisms, and Electronic Characterization of P3HT Films Doped with Various Systems. (a) Chemical structure of the TAB–2TFSI dopant and a schematic energy level diagram depicting the sequential doping mechanism. The proposed mechanism illustrates the thermodynamically feasible reduction of TAB^2+^ toward TAB^0^ by P3HT, followed by charge compensation with two TFSI^−^ anions. The corresponding chemical equation for the formation of 2[P3HT^+^·TFSI^−^] is provided. (b) Chemical structures and energy levels of benchmarking dopant systems. (c) UV–vis–NIR absorption spectra of pristine and doped P3HT films. The doping‐induced sub‐gap absorption features assigned to the P2 and P1 polaron bands appear in the red‐to‐near‐infrared region (≈700–1800 nm), while bleaching of the neutral absorption is observed in the visible range. (d) Comparison of electrical conductivities (*σ*) for P3HT thin films across various doping systems. The TAB–2TFSI doped film demonstrates superior performance compared to conventional benchmarks. Error bars represent the standard deviation calculated from three independent samples.

Figure 1 summarizes the design and doping mechanism of TAB–2TFSI (TAB^2+^·2TFSI^−^) and positions its performance against state‐of‐the‐art popular p‐dopants representing different doping mechanisms for semicrystalline P3HT. P3HT and TAB–2TFSI were synthesized in‐house following reported procedures (Section S1 and Scheme S1) [35]. Importantly, TAB–2TFSI can be prepared by a straightforward, scalable synthesis, supporting practical large‐scale implementation. The central distinguishing feature of TAB as a dopant is that it forms a dication, enabling either a stepwise reduction path (TAB^2+^ → TAB^+^ → TAB^0^) or a simultaneous double reduction path (TAB^2+^ → TAB^0^), and thus offering two pathways for two sequential polymer oxidation events from a single TAB molecule (Figure 1a). Considering the ionization energy of P3HT (≈−5.0 eV) and the redox energies associated with the reduction of TAB^2+^ and TAB^+^ (≈−5.67 and −5.57 eV, respectively; literature‐reported experimental/electrochemical values) [35], TAB–2TFSI can, in principle, generate two holes per dopant while reaching a neutral TAB^0^ final state. Density functional theory (DFT) calculations support this mechanism; both possible dopant reduction paths (stepwise and simultaneous) exhibit negative and comparable Gibbs free energy, confirming the thermodynamic viability of the two‐electron reduction of TAB^2+^ (Section S2 and Table S1). A key practical advantage of this architecture is that the dopant contains two TFSI^−^ anions that can directly provide charge compensation for the oxidized polymer segments, yielding a final ionic state that can be represented as 2[P3HT^+^·TFSI^−^] + TAB^0^ (overall stoichiometry shown in Figure 1a). In P3HT, such a double electron‐transfer step is not expected for the benchmark dopant F_4_TCNQ because reduction of F_4_TCNQ^−^ to F_4_TCNQ^2^^−^ corresponds to a much shallower energy level (*E*_redox_ ≈−4.7 eV; Figure 1b, left) than the P3HT ionization energy (≈ −5.0 eV). To contextualize this platform, we compared TAB–2TFSI with representative p‐dopant systems widely used for P3HT, including F_4_TCNQ, Magic Blue, and FeCl_3_ (Figure 1b). TAB–2TFSI can be compared with Magic Blue because both systems deliver the oxidizing species and counterion within a single‐compound dopant platform; however, TAB–2TFSI provides two TFSI^−^ counteranions instead of one, and the two systems differ substantially in their chemistry and molecular size. By delivering a moderately bulky, weakly coordinating TFSI^−^ counterion environment, TAB–2TFSI is designed to reduce carrier–ion coupling relative to more coordinating or smaller anions (such as SbCl_6_^−^ in Magic Blue). Reduced carrier‐ion coupling is expected to lead to enhanced charge delocalization and high mobility, along with a more stable ionic environment in the solid state [11, 34]. For fairness, we included in our study other popular and high‐performing anion‐exchange doping strategies involving TFSI: FeCl_3_/Li‐TFSI and F_4_TCNQ/Li‐TFSI protocols. A critical distinction between ion‐pair dopants like TAB–2TFSI and these conventional ion‐exchange systems lies in the initial state of the oxidizing species. While oxidation in the standard anion‐exchange protocols is driven by neutral/metal‐salt oxidants (e.g., FeCl_3_ or F_4_TCNQ), the oxidation in our system is driven by a pre‐formed, highly reactive dication (TAB^2^^+^). This dicationic nature provides a substantially stronger thermodynamic driving force for electron transfer, as evidenced by its exceptionally deep redox energy (*E*_redox_ ≈ −5.67 eV), fundamentally distinguishing its redox power from that of conventional oxidants. Finally, for a thorough comparison, we also included the recently‐reported F_4_TCNQ:BCF (BCF = tris(pentafluorophenyl)borane) system, as a next‐generation molecular‐doping benchmark, in which Lewis‐acid complexation modifies the effective doping strength and ion pairing of F_4_TCNQ‐based systems to enhance *σ* in semicrystalline polymer hosts [12, 38].

The UV–vis–NIR spectra corroborate successful p‐doping across the different dopant systems (Figure 1c). Film‐doping protocols and post‐treatments are provided in Section S3, with representative processing conditions summarized in Table S2a. Pristine P3HT exhibits the characteristic π–π* vibronic absorption in the visible region (≈450–650 nm, with a main band around ∼520 nm and vibronic structure toward ∼550–600 nm). Upon doping, this neutral absorption is strongly bleached and replaced by pronounced sub‐gap absorption features in the near‐infrared, including a broad band in the ≈700–1100 nm range and an additional contribution extending into the ≈1100–1800 nm region, consistent with the formation of polaronic sub‐gap absorption features, including the P2 and P1 polaron bands. Like the other popular doping systems, TAB–2TFSI produces a strong suppression of the neutral vibronic signature together with intense sub‐gap absorption throughout the near‐infrared, indicating efficient generation of oxidized P3HT segments in the solid state.

Electrical conductivity provides a direct performance benchmark for these doping strategies in semicrystalline P3HT (Figure 1d). Under comparable processing conditions (i.e., those that maximize conductivity) (Table S2a), TAB–2TFSI yields the highest average conductivity (290 ± 30 S cm^−^^1^), outperforming F_4_TCNQ, Magic Blue, and the traditional anion‐exchange protocols (FeCl_3_/Li‐TFSI and F_4_TCNQ/Li‐TFSI). To further connect this performance advantage to the doping state, we estimated the charge carrier density from the P2 polaron absorption at 800 nm (Figure 1c) and calculated the corresponding mobility using the measured conductivity (Table S2b). This optical analysis indicates that, among the high‐conductivity systems, TAB–2TFSI‐doped P3HT exhibits a comparable (slightly higher) charge‐carrier density but the highest mobility estimate, suggesting that its conductivity advantage is closely associated with more efficient charge transport rather than simply a higher carrier density. This motivates a closer examination of dopant‐induced microstructural changes in P3HT.

### 2.2 Structural Characterization of Doped P3HT Films

**FIGURE 2:**
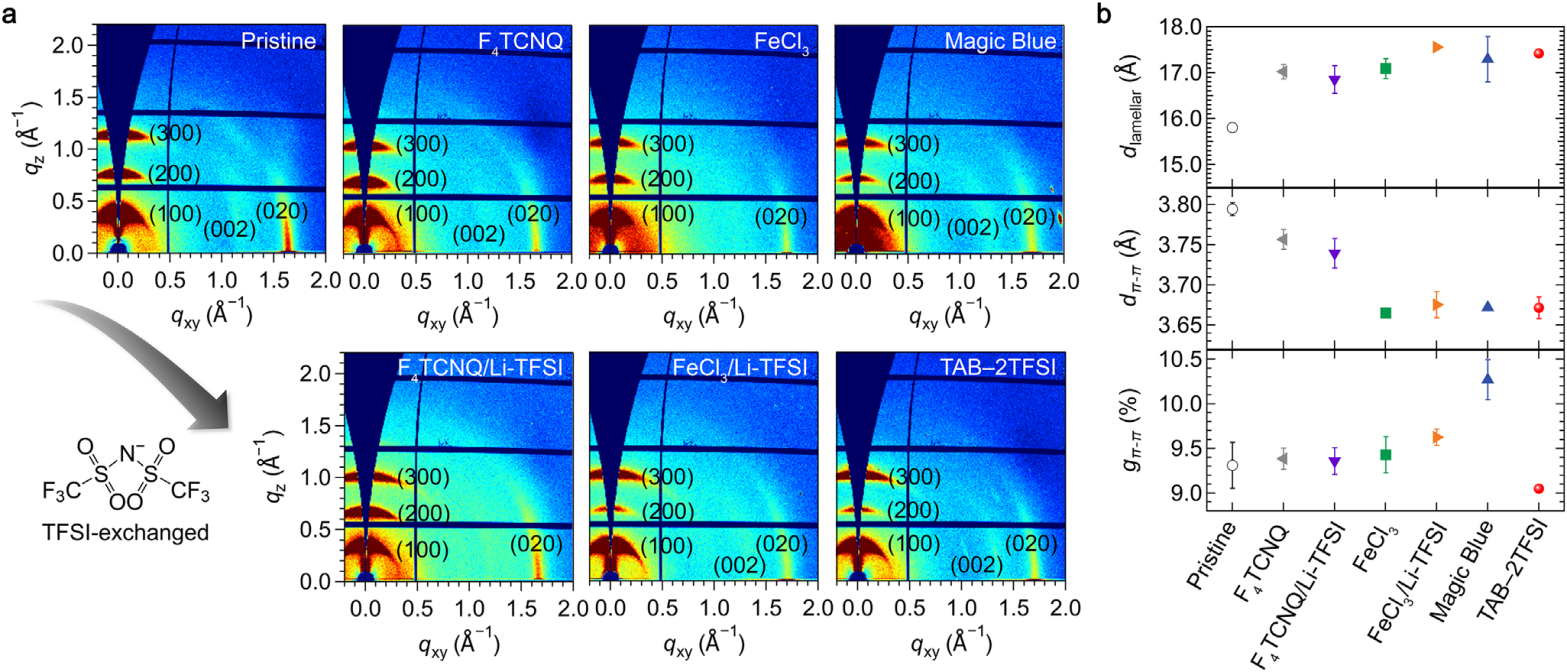
Structural analysis of P3HT films using GIWAXS. (a) 2D GIWAXS patterns of pristine P3HT and P3HT films doped with various dopants (F_4_TCNQ, FeCl_3_, and Magic Blue), their ion‐exchange TFSI‐incorporated counterparts (F_4_TCNQ/Li‐TFSI, FeCl_3_/Li‐TFSI), and TAB–2TFSI. (b) Structural parameters extracted from the GIWAXS data: lamellar spacing (*d*_lamellar_), stacking distance (*d*_π–π_), and the paracrystallinity factor (*g*_π–π_). Error bars represent the standard deviation calculated from three independent samples.

To examine how the different doping strategies modulate the semicrystalline packing of P3HT, we performed GIWAXS on pristine and doped films (Figure 2a). Additional GIWAXS details, including data processing and fitting procedures, are provided in Section S4 and Figure S1. All films shown in Figure 2a retain the characteristic features of semicrystalline P3HT, including lamellar stacking reflections ((*h*00) family) at low *q* and a π–π stacking reflection (020) at high *q* (1.66–1.71 Å^−1^, corresponding to *d*_π–π_ ≈ 3.79–3.67 Å). This result indicates that the dominant crystalline motif is preserved upon doping, although the lattice parameters and stacking order are significantly modified. In particular, quantitative peak analysis (Figure 2b and Table S3) reveals clear dopant‐dependent trends in the lamellar spacing *d*_lamellar_, π–π stacking distance *d*_π–π,_ and π–π paracrystallinity *g*_π–π_. The structural parameters summarized in Figure 2b were extracted from 1D linecut profiles of the 2D GIWAXS patterns; details about the corresponding line cuts and peak analyses are provided in Section S4; Figure S2 and Table S3. Relative to pristine P3HT (*d*_lamellar_ ≈ 15.8 Å, corresponding to *q*_100_ ≈ 0.40 Å^−1^), doping expands the lamellar spacing into the ∼16.9–17.6 Å range, suggesting that dopants are allocated at the side chains. Notably, the TFSI‐based systems show the largest lamellar expansion, with *d*_lamellar_ increasing to ∼17.3–17.6 Å across F_4_TCNQ/Li‐TFSI, TAB–2TFSI, and FeCl_3_/Li‐TFSI (Figure 2 and Table S3), consistent with the bulkier character of the counterion TFSI^−^ compared to F_4_TCNQ^−^ or SbCl_6_^−^. In parallel, π–π stacking contracts upon doping from *d*_π–π_ ≈ 3.79 Å (pristine, *q*_020_ ≈ 1.66 Å^−1^ Table S3) to ∼3.67–3.76 Å depending on the dopant. The contraction of π–π stacking, coinciding with the expansion of the lamella distance upon doping, has been widely reported. This structural evolution is primarily driven by the intercalation of dopant anions into the alkyl side‐chain domains, which helps to extend the lamellar spacing [5]. The most pronounced contraction is observed for TAB–2TFSI, FeCl_3_/Li‐TFSI, Magic Blue, and FeCl_3_, which converge to *d*_π–π_ ≈ 3.67–3.68 Å (*q*_020_ ≈ 1.71 Å^−1^, Table S3). Those dopants correspond to the most conductive samples shown in Figure 1d, which is not surprising because shorter π–π stacking distances are associated with easier intermolecular charge transport and higher electrical mobility. Notably, the F_4_TCNQ:BCF‐doped P3HT film shows severe peak broadening and a loss of distinct crystalline reflections (Section S5 and Figure S3).

Importantly, the π–π paracrystallinity, which has been used as a proxy for mobility in conjugated polymers, nuances the observed correlation between reduced π–π spacing and the high conductivity [18, 39]. Here, a lower *g*_π–π_ value corresponds to lower crystalline disorder, i.e., a narrower distribution of π–π stacking distances, and is therefore more favorable for charge transport. In particular, Magic Blue shows clearly higher disorder (*g*_π–π_ ≈ 10.27%) than TFSI‐based films (*g*_π–π_ ≈ 9.6% for FeCl_3_/Li–TFSI and *g*_π–π_ ≈ 9.05% for TAB–2TFSI) despite its comparable low *d*_π–π_ and high conductivity. A plausible interpretation for the lower crystalline order of Magic Blue compared to the other tested TFSI‐based films is that counterion identity and its spatial incorporation govern the distribution of local π–π stacking environments. In the Magic Blue system, the more strongly coordinating SbCl_6_^−^ counterion has been reported to reside predominantly in amorphous regions of semicrystalline P3HT [40], which can lead to a spatially heterogeneous ionic landscape, thereby increasing the apparent *g*_π–π_. This result suggests that, at least in these isotropic films, Magic Blue might compensate for its comparatively lower crystalline order with better inter‐crystallite connectivity, yielding still relatively high mobility (Table S2b). By contrast, TFSI^−^ is comparatively bulky and weakly coordinating, and can be more uniformly accommodated within both the amorphous and crystalline regions [41], generating lower π–π stacking distance variations. Notably, even within the TFSI^−^‐based systems, TAB–2TFSI yields a lower *g*_π–π_ than FeCl_3_/Li‐TFSI despite similar *d*_π–π_, indicating lower π–π stacking disorder and suggesting that the nanoscale uniformity of incorporated TFSI^−^ may be the highest for the TAB system. In particular, the exchange of two different anions in FeCl_3_/Li‐TFSI can be thermodynamically limited and potentially leave a mixed‐counterion environment [33], whereas TAB–2TFSI allows only TFSI^−^ as the counterion, which replaces a neutralized TAB^0^ molecule. This motivates a direct nanoscale comparison of the spatial distribution of incorporated TFSI^−^ in the two films.

**FIGURE 3:**
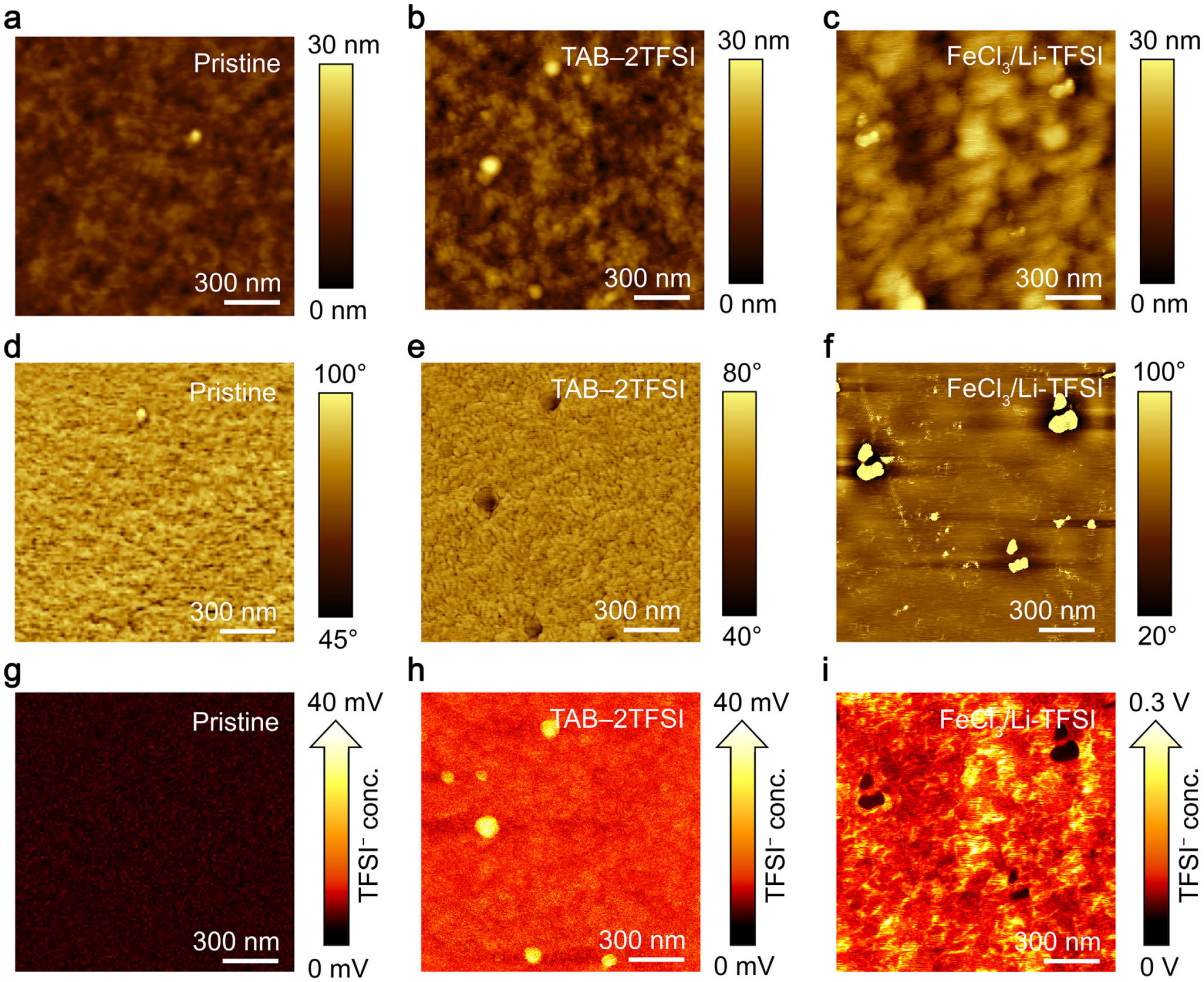
AFM‐IR characterization of pristine and doped P3HT films with spatial mapping of the TFSI‐related IR response. AFM topography (a–c), phase (d–f), and AFM‐IR maps acquired at 1350 cm^−1^ (g–i) are shown for pristine P3HT, TAB–2TFSI, and FeCl_3_/Li‐TFSI‐doped P3HT films. In AFM‐IR, the higher the voltage of the measurement, the higher the 1350 cm^−1^ signal. Pristine P3HT exhibits a negligible IR response (∼ 0 V), whereas TAB–2TFSI shows a broadly uniform TFSI‐related signal (≥20 mV) across the surface. In contrast, FeCl_3_/Li–TFSI displays pronounced spatial heterogeneity with localized high‐voltage (0.3 V) domains. The scale bars represent 300 nm.

Figure 3 summarizes the AFM‐IR measurements of pristine, TAB–2TFSI‐doped, and FeCl_3_/Li‐TFSI‐doped P3HT films, including topography (Figure 3a–c), phase (Figure 3d–f), and IR response maps (Figure 3g–i) acquired at 1350 cm^−1^ (read as voltage signals). The rationale for selecting 1350 cm^−^^1^ as a TFSI‐related marker band stems from the negligible IR response observed for pristine P3HT (Figure 3g) (see details in Section S6 and Figure S4). Contrary to the FeCl_3_/Li‐TFSI films, the TAB–2TFSI films displayed similar topography and phase maps as the pristine P3HT, indicating a minimal disturbance of the original micro/nanostructure. The TAB–2TFSI film exhibited a broadly uniform AFM‐IR signal (Figure 3h), whereas the FeCl_3_/Li‐TFSI film displayed localized high‐voltage domains (Figure 3i), which suggests a more heterogeneous distribution of TFSI‐related species in the FeCl_3_/Li‐TFSI film. The more uniform TFSI‐related response likely originates from the dicationic ion‐pair nature of TAB–2TFSI, which delivers the oxidizing TAB^2+^ unit and TFSI^−^ counterions in a single compound without requiring a separate Li‐TFSI‐mediated ion‐exchange step. Moreover, if TAB^2+^ reduces to TAB^0^, no movable cation remains in the system. In contrast, FeCl_3_/Li‐TFSI doping requires charge transfer from the polymer to the FeCl_3_ followed by ion exchange with TFSI^−^ from the added Li‐TFSI salt, and re‐association of the reduced version of FeCl_3_ compound with Li^+^. This complex mixed‐ion dynamics may leave localized TFSI‐rich domains if the exchange/re‐association process is incomplete. Because the 1350 cm^−1^ signal indicates TFSI‐containing species regardless of whether they act as a counterion or are present in residual salt, we conservatively assign the localized high‐voltage features in the FeCl_3_/Li‐TFSI film to TFSI‐rich local domains in general. In any case, these TFSI clusters are less effective for charge compensation in oxidized P3HT, and hence for efficient and stable doping. This TFSI cluster interpretation is supported by the overlap of the high‐voltage (yellow) regions in Figure 3i with slightly elevated areas in Figure 3c. Furthermore, TFSI‐rich domains may induce spatial electrostatic variations, potentially contributing to a broader distribution of local π–π stacking distances. This observation aligns with the GIWAXS study in Figure 2b, revealing higher paracrystallinity for FeCl_3_/Li‐TFSI than for TAB–2TFSI, and with the *σ* trend in Figure 1d. For both analyzed doping systems, the phase pattern does not overlap with the IR color map. Assuming the AFM phase contrast originates from crystallinity differences, the lack of correlation with IR color suggests that the dopant distributes homogeneously in P3HT without favoring crystalline or amorphous regions. This is consistent with observations reported in previous studies [40].

These combined GIWAXS and AFM‐IR results indicate that TAB–2TFSI morphology, namely tight π–π stacking distance, low π–π disorder, and a homogeneous TFSI‐related ionic landscape, is ideal for charge transport. In the next section, we examine how these structural signatures correlate with TE performance.

### 2.3 Charge Transport and Thermoelectric Performance of Doped P3HT Films

**FIGURE 4:**
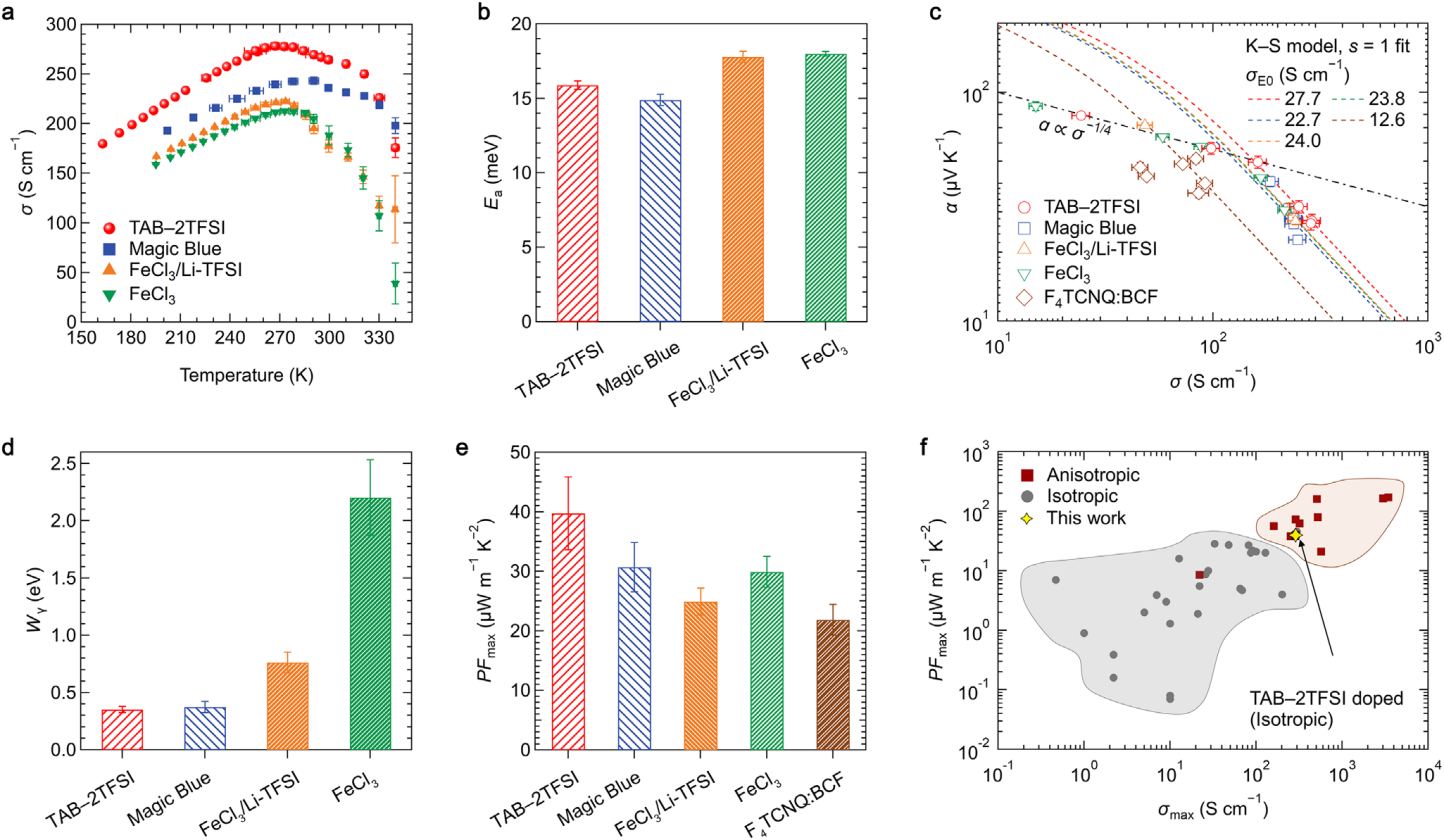
Charge transport and thermoelectric properties of doped P3HT films. (a) Temperature‐dependent electrical conductivity *σ*(*T*) of P3HT films doped with TAB–2TFSI (red), Magic Blue (blue), FeCl_3_/Li–TFSI (orange), and FeCl_3_ (green) from 150 to 350 K. (b) Comparison of the activation energy *E*_a_ extracted from *σ*(*T*). (c) Seebeck coefficient (*α*) as a function of *σ*. The dashed lines represent fits based on the Kang–Snyder (K–S) model (*s* = 1) with different transport edge parameters (*σ*_E0_). (d) Extracted energetic disorder (*W*_γ_) for the various doping systems, indicating the reduced disorder in TAB–2TFSI‐doped films. (e) Maximum thermoelectric power factors (*PF*_max_) of P3HT films doped with different systems. (f) Benchmarking of *PF*_max_ versus maximum electrical conductivity *σ*_max_ of TAB–2TFSI‐doped P3HT (yellow star) against previously reported isotropic (grey) and anisotropic (brown) P3HT systems. Error bars for room‐temperature thermoelectric parameters represent the standard deviation from three independently prepared samples. For the temperature‐dependent transport and Kang–Snyder analyses, uncertainties in the extracted parameters were obtained from fitting uncertainty and error propagation, as described in the Supporting Information.

We next examined the temperature‐dependent conductivity *σ*(*T*) and TE response of the most conductive doped P3HT films (Figure 4). Measurement details and analysis procedures are described in Section S7. Across all doped samples, *σ* exhibited a non‐monotonic temperature dependence: *σ* increased upon heating at low temperature and then decreased above a characteristic temperature, indicating a crossover between thermally assisted transport and a metal‐like regime where additional phonon scattering effects suppress conductivity at higher temperature (Figure 4a). Notably, the TAB–2TFSI‐doped film showed this turnover at a lower temperature (≈ 267 K) than for the FeCl_3_‐based systems (≈273 K) and Magic Blue (≈290 K). This behavior is consistent with an effectively percolated conductive network [42].

To quantify the degree of thermal activation required for charge transport, we extracted an apparent activation energy (*E*_a_) from the temperature‐dependent conductivity (Figure 4b; Section S7 and Figure S5a). The corresponding temperature‐dependent Seebeck coefficient, *α*(*T*), is provided in the Supporting Information (Figure S5b). TAB–2TFSI and Magic Blue display similarly low *E*_a_ values (≈15–16 meV) compared to FeCl_3_/Li–TFSI and FeCl_3_ (≈17.8–18 meV), suggesting that the dominant transport pathways in these two systems require a smaller thermal energy to conduct. To further quantify dopant‐dependent transport beyond the Arrhenius activation picture, we analyzed the Seebeck–conductivity relationships of the doped films using the Kang–Snyder (K–S) framework (Figure 4c) [43]. The room‐temperature *α*–*σ* datasets used for this analysis were obtained from the home‐built setup (Section S8, Figures S6 and S7; Table S4), and details of the K–S analysis framework are provided in Section S9. The measured *α*–*σ* datasets are well described by the *s* = 1 K–S model, associated with delocalized transport, particularly for the more heavily doped (low‐*α* high‐*σ*) samples, whereas at lower doping levels the datasets deviate toward an *α* ∝ *σ* ^−1/4^ trend that is commonly associated with more localized transport [44, 45]. Within the *s* = 1 regime, the transport coefficient *σ*_E0_ differs systematically across dopants, and TAB–2TFSI yields the highest *σ*_E0_ (27 S cm^−1^) among the compared systems, suggesting enhanced effective transport in the conducting network (often interpreted as reflecting higher effective mobility). Interestingly, even the most conducting Magic Blue‐doped films fabricated displayed a considerably lower *σ*_E0_ (∼22.7 S cm^−1^), bolstering our morphology‐inferred claims of comparatively lower mobility. These results are consistent with the mobility extracted from UV‐Vis spectroscopy (Table S2b). To further link these transport descriptors to microstructural connectivity, we evaluated the transport‐barrier parameter *W_γ_* extracted from the temperature dependence of *σ*_E0_, obtained from the measured *σ*(*T*) and *α*(*T*) datasets (Figure 4d; Section S9 and Figure S8). TAB–2TFSI exhibits the lowest *W*_γ_ value (≈0.35 eV), comparable to Magic Blue (≈0.37 eV), and significantly smaller than FeCl_3_/Li‐TFSI (≈0.76 eV) and FeCl_3_ (≈2.2 eV), indicating a reduced energetic barrier for charge transport between conducting domains [46]. This reduction is consistent with a more continuous transport network and diminished inter‐domain bottlenecks. It is important to indicate that while *E_a_* and *W_γ_* provide indirect information on the material order, such information is complementary to that provided by GIWAXS. This is due to the very different nature of the measurements, and the fact that GIWAXS considers only crystalline regions, while *W_γ_* offers insights into inter‐domain transport (i.e., the tie‐chains in the amorphous region connecting crystalline domains), and *E_a_* provides insights into the overall transport mechanism encompassing both amorphous and crystalline regions [9]. As a result of the combined improvements in intrinsic transport parameters (high *σ*_E0_) and reduced inter‐domain barriers (low *E_a_* and *W*_γ_), TAB–2TFSI delivers the highest maximum average power factor (*PF*_max_) (40 ± 6 µW m^−1^ K^−2^) at a concentration of 0.5 mM among the investigated dopant systems (Figure 4e; Figure S7 and Table S4). Such high *PF* places TAB–2TFSI‐doped P3HT in a *σ*–*PF* plot above representative reported isotropic polymer thermoelectrics (Figure 4f), in a region so far reserved for anisotropic aligned materials. Here, anisotropic P3HT refers to films subjected to deliberate macroscopic chain‐alignment processes, such as high‐temperature rubbing, whereas isotropic P3HT denotes non‐aligned films, such as the spin‐coated films used in this work. Because chain alignment can markedly enhance direction‐dependent charge transport and *PF* but requires additional processing, achieving this level of performance in non‐aligned films highlights the effectiveness of TAB–2TFSI doping.

### 2.4 Ambient and Thermal Stability of Doped Films

**FIGURE 5:**
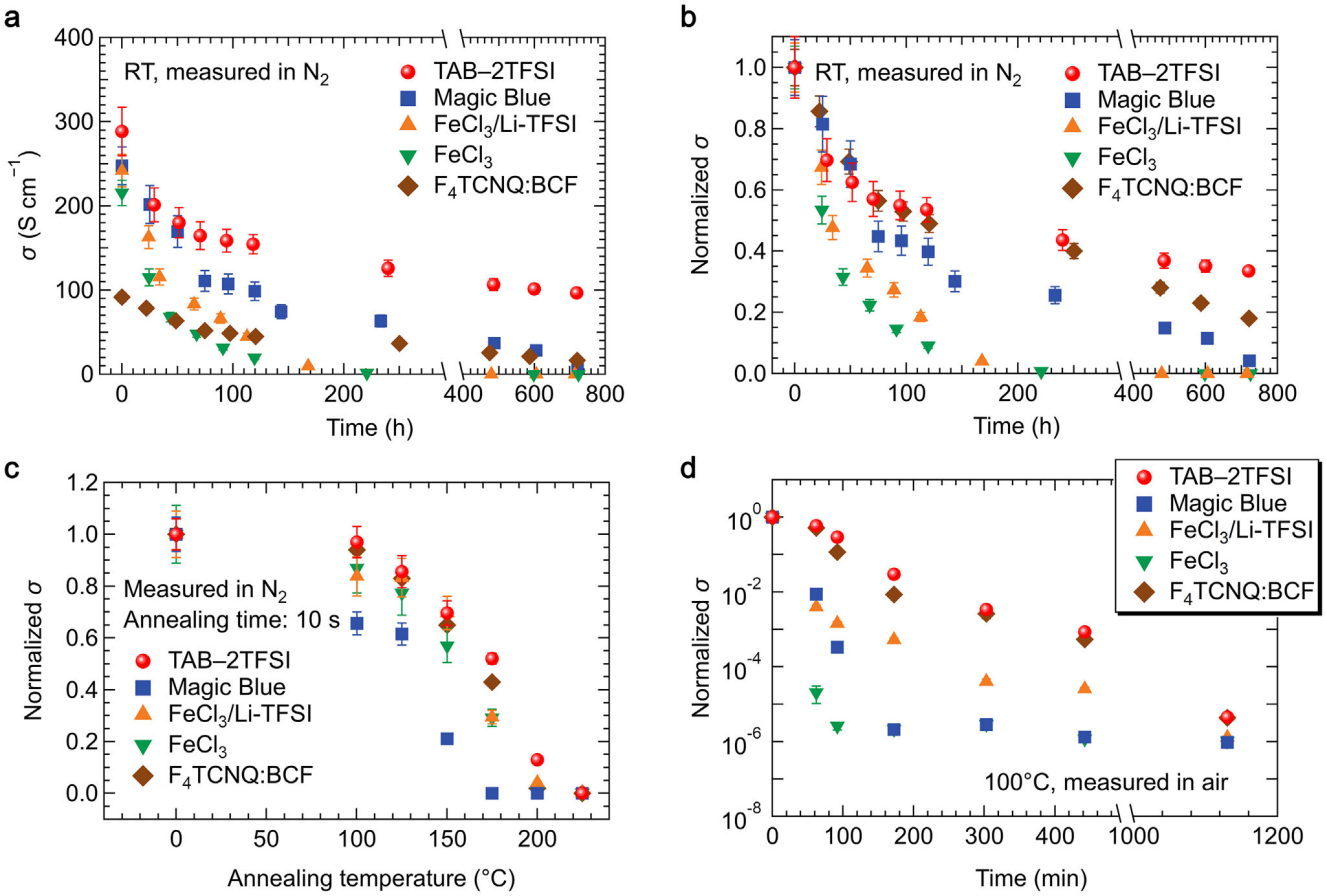
Thermal and ambient stability of doped P3HT films. (a) Temporal stability of *σ* and (b) corresponding normalized electrical conductivity (*σ*/*σ*_0_) of P3HT films doped with various dopant systems, stored at room temperature (RT) in a nitrogen (*N*_2_) atmosphere for over 700 h. (c) Thermal stability of the doped films, represented by normalized electrical conductivity measured at RT after 10 s of annealing at various temperatures ranging from RT to 225°C. (d) Accelerated ambient degradation test showing normalized electrical conductivity over time for doped films exposed to ambient air at a constant temperature of 100°C. Error bars represent the standard deviation calculated from three independent samples.

To assess the robustness of the doped state under conditions relevant to OTE, we monitored the time‐ and temperature‐dependent stability of doped P3HT films under inert and oxidative environments (Figure 5). To evaluate the most adverse scenario for stability, the samples presenting maximum conductivity, and therefore the highest doping level, were tested, instead of those presenting the maximum *PF* (usually at moderate doping levels). First, we tracked *σ* as a function of aging time at room temperature under *N*_2_ (Figure 5a). Across all dopant systems, *σ* decreases with time even in an inert atmosphere, indicating that the doped state continues to evolve after processing. Notably, TAB–2TFSI‐doped films retain the highest *σ* over extended aging times, whereas FeCl_3_‐ and FeCl_3_/Li‐TFSI ‐doped films exhibit a more pronounced decay. The same trend is emphasized when the data are plotted in normalized form (Figure 5b), highlighting the comparatively improved retention of TAB–2TFSI relative to the benchmark dopants despite its higher nominal conductivity. After ∼90 h of aging in N_2_, TAB–2TFSI retains ∼55% of its initial conductivity, a stability only contended by F_4_TCNQ:BCF (∼53%), which was recently reported as the most stable dopant system [12], and Magic Blue (∼43%); whereas the FeCl_3_‐based systems show substantially faster decay (FeCl_3_/Li–TFSI: ∼27%; FeCl_3_: ∼15%). Consistent with these trends, TAB–2TFSI and F_4_TCNQ:BCF remain above 50% retention until **∼**120 h, while Magic Blue falls below 50% between **∼**50–80 h and the FeCl_3_/Li–TFSI and FeCl_3_ samples cross the same threshold within ∼30–50 h and 15–30 h, respectively. Complementary time‐stability data for the Seebeck coefficient and power factor measured under N_2_ are provided in Section S10 and Figure S9. Consistent with *σ* decay, *α* generally increases upon aging under N_2_ (Figure S9a), suggesting a gradual reduction in the effective doping level and charge carrier density. Thus, the resulting *PF* retention depends strongly on the dopant system (Figure S9b). Among the compared systems, TAB–2TFSI maintains a high *PF* upon aging under N_2_, retaining 17 ± 1 µW m^−^^1^ K^−^^2^ after 720 h (from 22 ± 3 µW m^−^^1^ K^−^^2^ initially; ∼80% retention) (Figure S9b). Notably, the P3HT samples moderately doped with TAB–2TFSI (0.5 mM), which yielded the maximum initial *PF* (Figure S7), also showed superior long‐term stability. This sample retained a *PF* of 23 ± 2 µW m^−^^1^ K^−^^2^ after 30 days (720 h) of nitrogen aging, compared to its initial value of 40 ± 6 µW m^−^^1^ K^−^^2^. Interestingly, an ACN‐rinsing control experiment confirmed that this long‐term decay under N_2_ is not dominated by removable residual dopant species: rinsed and unrinsed TAB–2TFSI films exhibited nearly identical normalized conductivity retention within experimental uncertainty (Figure S10).

We next evaluated thermal tolerance under inert conditions by briefly annealing the films (10 s) at increasing temperatures in N_2_ and measuring *σ* (Figure 5c). TAB–2TFSI shows the most robust retention across the annealing, maintaining ∼97% at 100°C and ∼70% at 150°C (normalized *σ*), followed by F_4_TCNQ:BCF, whereas Magic Blue drops more strongly (to ∼65% at 100°C and ∼21% at 150°C). Above 175°C all systems degrade rapidly, although TAB–2TFSI still retains 52% of its initial conductivity after annealing at 175°C, before approaching near‐zero by ∼225°C. Finally, to probe combined thermal and oxidative stress relevant to device operation, we aged the doped films at 100°C in air and monitored *σ* decay (Figure 5d). Under these harsher conditions, *σ* decreases by multiple orders of magnitude for all systems; nevertheless, TAB–2TFSI and F_4_TCNQ:BCF exhibit comparatively slower decay, remaining around ∼10^−^^3^ after ∼300–450 min and ∼10^−^^5^ after ∼1200 min, whereas Magic Blue and FeCl_3_ rapidly collapse to ∼10^−^^6^ within the first few hours. Collectively, these aging and thermal stress tests show that TAB–2TFSI not only provides the highest conductivity, but together with F_4_TCNQ:BCF, it also provides the most robust retention of *σ* across inert storage, thermal annealing, and oxidative aging conditions.

**FIGURE 6:**
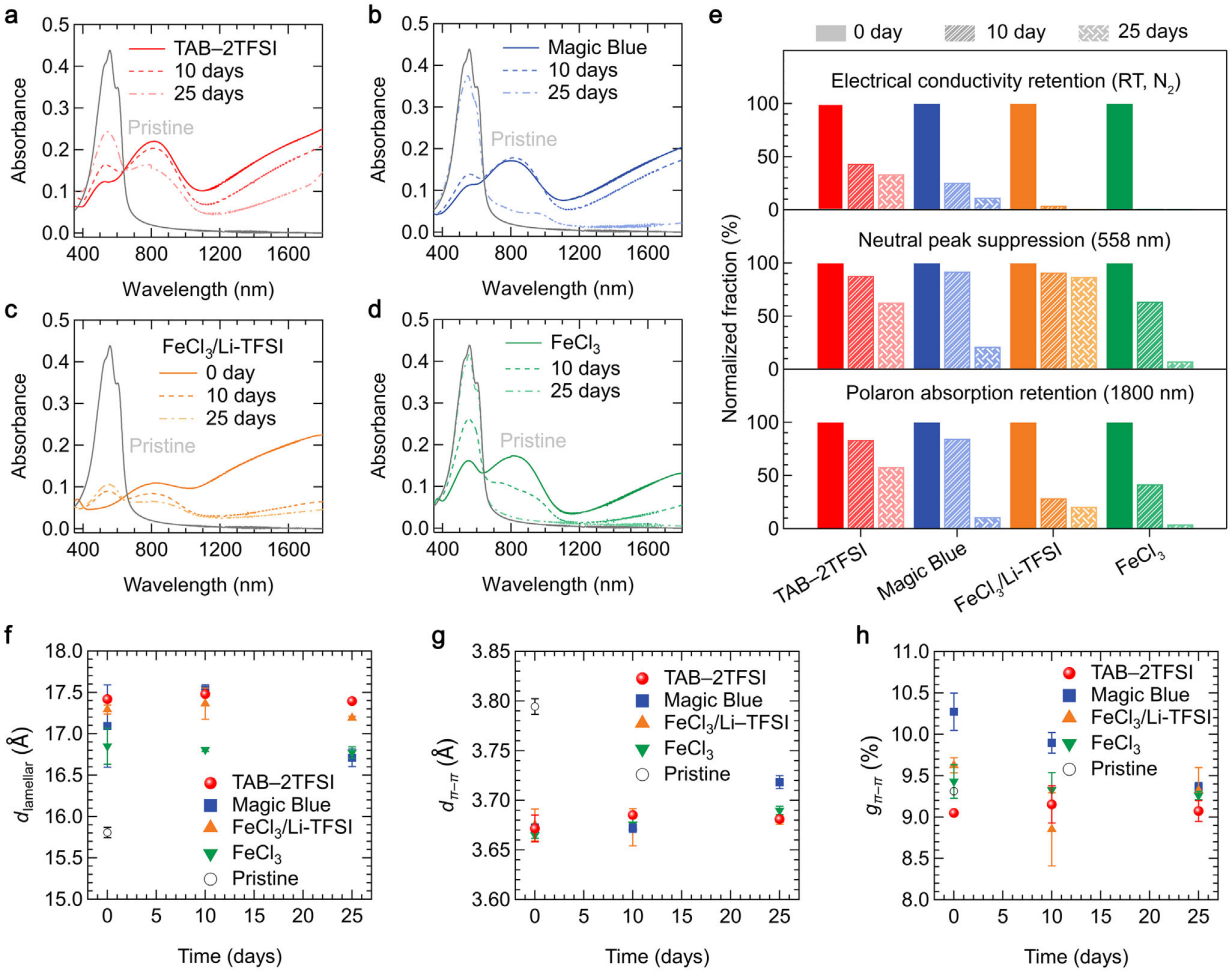
Optical and structural stability analysis of doped P3HT films during long‐term storage. (a–d) Time‐dependent UV–vis–NIR absorption spectra of P3HT films doped with (a) TAB–2TFSI, (b) Magic Blue, (c) FeCl_3_/Li‐TFSI, and (d) FeCl_3_ measured after 0, 10, and 25 days of storage in N_2_ atmosphere. Gray lines show the pristine P3HT spectrum for reference. (e) Electrical conductivity retention (RT, N_2_), neutral peak suppression at 558 nm, and polaron absorption retention at 1800 nm, each normalized to the 0‐day value for the corresponding dopant system (0 day = 100%). (f–h) Evolution of GIWAXS‐derived structural parameters after 0, 10, and 25 days of storage in a N_2_ atmosphere: (f) *d*_lamellar_, (g) *d*_π–π_, and (h) *g*_π–π_. Error bars represent the standard deviation calculated from three independent samples.

To assess how doping‐state and structural evolution contribute to *σ* decay (Figure 5), we monitored the time evolution of the optical doping signatures and semicrystalline packing of the most conductive doped P3HT films (Figure 6). UV–vis–NIR spectra collected at 0, 10, and 25 days reveal strongly dopant‐dependent stability of the polaronic sub‐gap absorption at *λ* = 1800 nm, and the neutral vibronic peak at *λ* = 558 nm (Figure 6a–d). For TAB–2TFSI, the polaron‐related sub‐gap absorption remains comparatively robust while the recovery of the neutral vibronic features is modest over time (Figure 6a), consistent with a more stable doped state. In contrast, the benchmark dopants exhibit substantially larger spectral drift (Figure 6b–d). Magic Blue‐ and FeCl_3_‐doped films showed a pronounced recovery of the neutral vibronic absorption accompanied by a strong attenuation of the sub‐gap polaronic features over aging (Figure 6b,d), consistent with extensive de‐doping. FeCl_3_/Li–TFSI‐doped films behave differently: while the neutral vibronic signature does not fully recover, the polaronic sub‐gap absorption decreases markedly with aging (Figure 6c), suggesting that aging primarily suppresses the polaronic contribution rather than fully restoring the neutral absorption. These trends are summarized in Figure 6e, which reports the retention of electrical conductivity (*σ*/*σ*_0_) together with absorbance‐based metrics: neutral vibronic peak suppression at *λ* = 558 nm, (*A*_λ, pristine_ − *A*_λ, t_) / (*A*_λ, pristine_ − *A*_λ, 0_), and polaron absorption retention at *λ* = 1800 nm, *A*_λ, t_/*A*_λ, 0_; each quantity is normalized to its own 0‐day value (0 day = 100%). Here, *A*_λ, t_ is the absorbance at wavelength *λ* after aging time *t*. TAB–2TFSI retains the highest fractions across these metrics, consistent with its superior stability of electrical performance during aging. The UV–vis–NIR aging spectra of the F_4_TCNQ:BCF‐doped films are shown separately in Figure S11.

While these optical trends indicate dopant‐dependent evolution of the electronic doping signatures, *σ* can also be governed by the changes in the semicrystalline packing. To probe this structural contribution, we performed time‐dependent GIWAXS on films stored under N_2_ and extracted GIWAXS parameters as a function of time (Figure 6f–h; Section S11 and Figures S12 and S13). The 2D GIWAXS maps confirm that the semicrystalline diffraction features of the doped P3HT films are largely preserved over aging, with only modest changes in scattering intensity and peak shape across 0–25 days (Figure S12). By contrast, the F_4_TCNQ:BCF‐doped films show diffuse scattering without distinct peaks throughout aging (Figure S14), which deserves further study. Consistent trends are evident in the corresponding 1D line‐cut profiles, where the (020) in‐plane reflections and the (100) out‐of‐plane lamellar peaks can be directly compared across time (Figure S13). Quantitatively, the extracted parameters—*d*_lamellar_, *d*_π–π_, and *g*_π–π_—show dopant‐specific temporal evolution (Figure 6f–h). In contrast to its comparatively stable counterparts, Magic Blue shows the most pronounced structural drift, featuring a large decrease in *d*_lamellar_ from ∼17.2 Å to ∼16.7–16.8 Å (Δ ≈ −0.4 to −0.5 Å) together with an increase in *d*_π–π_ from ∼3.67 Å to ∼3.72 Å (Δ ≈ +0.04–0.05 Å) by day 25. This is accompanied by a notable reduction in *g*_π–π_ (from ∼10.3% to ∼9.3%; Δ ≈ −1.0%), consistent with substantial time‐dependent reorganization of the semicrystalline packing. TAB–2TFSI exhibits the smallest temporal evolution, with *d*_lamellar_ remaining essentially constant at ∼17.4–17.5 Å (Δ ≲ 0.1 Å) and *d*_π–π_ staying near ∼3.67–3.68 Å (Δ ≈ 0.01 Å) from day 0 to day 25. In addition, *g*_π–π_ is also well preserved for TAB–2TFSI (∼9.1% throughout; Δ ≲ 0.1%), indicating minimal change in π–π packing disorder during aging. Notably, although TAB–2TFSI shows minimal temporal evolution in GIWAXS‐derived packing parameters, AFM‐IR mapping at the TFSI‐related marker band (1350 cm^−^^1^) reveals increased spatial contrast after 25 days of inert aging (Figure S15). This suggests residual nanoscale redistribution/segregation of TFSI‐related species, likely occurring predominantly in amorphous‐rich regions that are less reflected in GIWAXS, and may contribute to the remaining *σ* decay even when the average semicrystalline packing is largely preserved. This result suggests that increasing crystallinity could be a route to increase the ambient stability of *σ*.

Taken together, the optical and GIWAXS results—supported by AFM–IR mapping (Figure S15)— indicate that TAB–2TFSI better stabilizes both the electronic doping signatures and semicrystalline packing order, providing a mechanistic basis for its superior conductivity retention observed in Figure 5. The reason for this stabilization, when comparing to Magic Blue and FeCl_3_/Li‐TFSI, could lie in a combination of counterion kinetics and dopant redox mechanism: On one hand, relative to FeCl_3_/Li‐TFSI, the process of a neutral‐ion replacement in TAB–2TFSI may avoid the more heterogeneous ionic/electrostatic environment and possible inefficiencies associated with a dual‐ion exchange process in ion‐exchange systems. Avoiding this mixed‐ion environment may help minimize local electrostatic perturbations, which could contribute to the slower decay of polaronic species in TAB–2TFSI compared with the faster decay observed in the FeCl_3_/Li‐TFSI (Figure 6). Additionally, our Gibbs free energy analysis does not exclude that TAB–2TFSI undergoes a simultaneous two‐electron transfer (Table S1). This potential concerted process might introduce a larger kinetic or thermodynamic barrier for de‐doping than a single‐electron transfer, offering a supplementary stabilization pathway. On the other hand, when comparing to Magic Blue, the bulkier character of TFSI^−^ compared to SbCl_6_^−^ might hinder diffusion during aging, stabilizing the polymer doping (especially in the crystalline region). This hypothesis seemed to be supported by the contraction of the lamella distance upon aging measured in Magic Blue but not in TFSI‐based dopants (Figure 6f), suggesting faster depletion or redistribution of counterion SbCl_6_^−^ from the side chain region. We next ask whether these combined benefits extend beyond the model system P3HT.

### 2.5 Generality of TAB–2TFSI Across Diverse Conjugated Polymers

**FIGURE 7:**
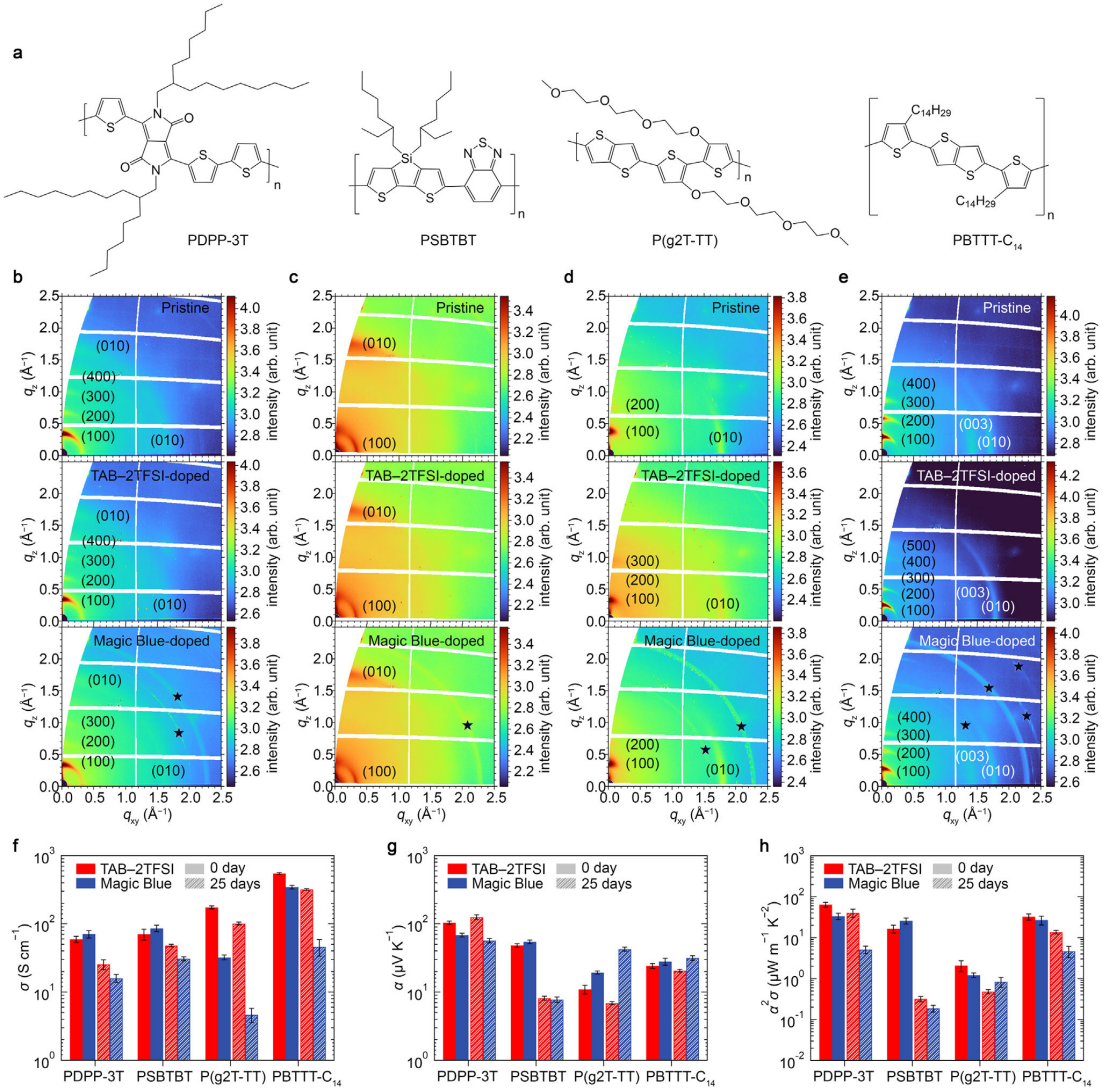
General applicability of TAB–2TFSI across diverse conjugated polymers. (a) Molecular structures of the four tested conjugated polymers (left to right: PDPP‐3T, PSBTBT, P(g2T‐TT), and PBTTT‐C_14_). (b–e) 2D GIWAXS patterns of polymer films in the pristine state (top), after doping with TAB–2TFSI (1.5 mM; middle), and after doping with Magic Blue (0.5 mM; bottom). Reflections associated with the lamellar spacing are indexed as (*h*00) and π–π stacking as (010); the (003) reflection corresponds to backbone longitudinal packing within individual π‐stacks. Black star symbols represent the Scherrer‐ring scattering from excess, unreacted Magic Blue dopant remaining on the film surface. (f–h) Time‐dependent evolution of TE parameters (*σ*, *α* and *σα^2^*) for polymers doped with TAB–2TFSI (red) and Magic Blue (blue), shown for as‐doped films (solid bars, 0 days) and after 25 days of aging at room temperature and under an inert atmosphere (hatched bars). Error bars represent the standard deviation from three independently prepared films.

To assess whether the stability and performance benefits of TAB–2TFSI observed in P3HT extend to other semicrystalline conjugated polymers, we expanded our study to a structurally diverse set of materials spanning different backbone motifs and side‐chain chemistries: the low‐bandgap donor–acceptor polymer PDPP‐3T, the silicon‐bridged dithienosilole–benzothiadiazole donor–acceptor polymer PSBTBT, the glycolated polythiophene P(g2T‐TT), and the highly planar, interdigitated polymer PBTTT–C_14_ (Figure 7a). TAB–2TFSI was used as the primary dopant, and Magic Blue was selected as a benchmark ion–pair dopant [47, 48], motivated by the fact that these two dopants deliver strong TE performance at least in the model system P3HT and the popular PBTTT, enabling a consistent comparison across polymer classes.

Figure 7b–e show representative 2D GIWAXS patterns for each polymer in the pristine state and after doping with TAB–2TFSI or Magic Blue. Across all four polymers, the doped films largely retain the characteristic diffraction features of the pristine films, indicating that the dominant semicrystalline motif is preserved upon doping. To quantify more subtle dopant‐induced structural changes, we extracted the peak positions and derived the corresponding lattice parameters, which are summarized in Table S5 (analysis procedures in Section S12). As was the case for P3HT, TAB–2TFSI induced a consistent reorganization of the lattice parameters: (i) systematic expansion of the lamellar spacing and (ii) contraction of the π–π stacking distance. Quantitatively, the lamellar spacing increased by ∼0.7–3.3 Å, while the π–π stacking distance decreased by ∼0.07–0.19 Å, depending on the polymer (Table S5). In several cases, the paracrystallinity parameter remained unchanged or slightly decreased, suggesting that the dopant‐driven lattice changes occurred without compromising crystallinity, and in some cases, may have reflected a modest improvement in structural order. By comparison, Magic Blue produced smaller changes than TAB–2TFSI in the lamellar and π–π stacking distances under comparable conditions (Figure 7b–e and Table S5).

We next examined whether these common structural trends correlate with TE performance and time‐dependent stability. Figure 7f–h and Table S6 summarize *σ*, *α*, and *α* ^2^ *σ* measured immediately after doping (0 day) and after 25 days of aging at room temperature under an inert atmosphere. In PDPP‐3T, TAB–2TFSI yielded an initial conductivity of 60 ± 10 S cm^−^^1^, comparable to that of Magic Blue. However, TAB–2TFSI shows better conductivity retention (26 ± 4 S cm^−^^1^) than Magic Blue (16 ± 2 S cm^−^^1^) after 25 days. In PSBTBT, both dopants achieved high initial *σ* (>70 S cm^−^^1^), yet TAB–2TFSI showed improved *σ* retention upon aging (67% versus 36% for Magic Blue). In the glycolated polymer P(g2T‐TT), TAB–2TFSI reached *σ* ≈ 175 S cm^−^^1^ and retained 58% of its initial conductivity after 25 days, outperforming the Magic Blue‐doped counterpart, consistent with a stabilizing contribution, also from polar side‐chain–counterion interactions. Finally, in highly crystalline PBTTT‐C_14_, TAB–2TFSI delivered high conductivity (*σ* ≈ 548 S cm^−^^1^) and superior long‐term retention, retaining 62% (*σ* ≈ 321 S cm^−^^1^) after 25 days—approximately matching the fresh Magic Blue benchmark (*σ* ≈ 347 S cm^−^^1^). Consistent with *σ* trends, the aging behavior across all four polymers is accompanied by attenuation of polaronic sub‐gap absorption and partial recovery of neutral absorption features (Figure S16), indicative of gradual de‐doping, with TAB–2TFSI generally exhibiting reduced spectral drift relative to the benchmark. The Seebeck coefficient remained comparable for both dopants before and after aging for all polymers, and remained similar or decreased after aging, except for P(g2T‐TT), for which Magic Blue outperformed TAB–2TFSI, and Seebeck increased with time. This trend, together with the reduction of *σ*, indicates a strong reduction in charge carrier density upon aging. In PDPP‐3T, TAB–2TFSI yielded the best *PF* measured in this study, with a peak power factor of 65 ± 9 µW m^−^^1^ K^−^^2^, approximately twice that obtained with Magic Blue (33 ± 6 µW m^−^^1^ K^−^^2^). Notably, TAB–2TFSI‐doped films retained ∼63% of their initial power factor after 25 days (41 ± 9 µW m^−1^ K^−2^), whereas the Magic Blue‐based reference retained ∼15%, indicating substantial performance drift. Overall, these results demonstrate that TAB–2TFSI is broadly applicable across chemically diverse semicrystalline polymers. It consistently tightened π–π stacking while preserving the characteristic diffraction signatures of the pristine films and improving TE stability compared to a representative ion–radical dopant benchmark, supporting the generality of the TAB–2TFSI dopant platform.

## 3 Conclusions

In summary, we demonstrate TAB–2TFSI, a dicationic ion‐pair dopant platform enabling simultaneously top TE performance and unprecedented operational stability compared to state‐of‐the‐art dopant systems in many semicrystalline conjugated polymers. By pairing a strong oxidizing motif with moderately bulky and weakly coordinating TFSI^−^ counterions, TAB–2TFSI enables a convenient balance between efficient doping, low ion‐charge interaction, minimal perturbance of the molecular structure upon doping, and a uniform counterion environment, all while establishing a stable ionic environment with reduced counterion‐driven microstructural evolution that can degrade doped films. This balance was confirmed by a thorough structure–transport analysis including theoretical calculations of Gibbs free energy, UV–vis spectroscopy, AFM‐IR, synchrotron GIWAXS, and measurements of thermoelectric properties versus temperature. Finally, the successful applicability of TAB–2TFSI across multiple conjugated polymer backbones highlights dicationic ion‐pair doping as a versatile and scalable strategy toward stable, high‐performance organic thermoelectrics. Optimized films yielded a *PF* of 40 ± 6 µW m^−^^1^ K^−^^2^ for P3HT, which decreased to 23 ± 2 µW m^−^^1^ K^−^^2^ after aging 720 h (30 days) in nitrogen and ambient light conditions, and 65 ± 9 µW m^−^^1^ K^−^^2^ for PDPP‐3T, which became 41 ± 9 µW m^−1^ K^−2^ after 25 days of aging in similar conditions. These values place TAB–2TFSI‐doped polymers as top thermoelectric performers among doped conjugated polymers, becoming competitive even with anisotropic polymers in terms of *PF*, and outperforming any previously reported materials in terms of stability.

Despite the significantly improved operational stability introduced by TAB–2TFSI, our findings also point toward subtle, long‐term ion‐redistribution processes that warrant further investigation. While GIWAXS analysis suggests that the long‐range crystalline order remains largely intact over time, AFM‐IR imaging (Figure S15) reveals a decrease in the uniformity in the distribution of TFSI in the P3HT film, suggesting that the amorphous phases might be more susceptible to de‐doping and that increased stability might be achieved by increasing crystallinity. The insight of this study might not only be decisive toward achieving truly ‘drift‐free’ performing conducting polymers, but it will contribute to shifting the focus of the community when it comes to designing molecular dopants: from simply targeting charge‐transfer efficiency to the holistic management of the dopant–host interface. Future work will explore the integration of TAB–2TFSI‐doped conducting polymer films into optimized thermoelectric modules to evaluate device‐level output under realistic temperature gradients. Ultimately, mastering dopant–host interaction offers a universal strategy to enhance the reliability of diverse organic electronic platforms, beyond thermoelectrics, for long‐term practical applications.

## 4 Experimental Section/Methods

### 4.1 Materials

P3HT and TAB–2TFSI were synthesized following previously reported procedures; detailed synthetic protocols and characterization are provided in Section S1. PBTTT‐C_14_ was purchased from Sigma–Aldrich. PSBTBT, P(g2T‐TT), and PDPP‐3T were purchased from TCI. Ion‐exchange salt Li‐TFSI (>99%, <1% water) was purchased from Sigma–Aldrich. Dopants F_4_TCNQ (>99%), FeCl_3_ (anhydrous, >98%, trace metals basis), and Magic Blue (technical grade) were purchased from Ossila and TCI. Anhydrous acetonitrile (Romil Hi‐Dry, <20 ppm water) was used to prepare all doping solutions, while anhydrous dichlorobenzene (Romil Hi‐Dry, <20 ppm water) was used for polymer solution preparation; further details are given below. All materials were used as received.

### 4.2 Solution Preparation

Solutions of P3HT were prepared in 1,2‐dichlorobenzene (o‐DCB) at 10 mg mL^−1^ and heated at 65°C overnight prior to use. Solutions of PBTTT‐C_14_, PSBTBT, P(g2T‐TT), and PDPP‐3T were prepared at the same concentration (10 mg mL^−^^1^) and heated at 60°C for at least 3 h prior to use. Dopant solutions were prepared in anhydrous acetonitrile (ACN) at the component concentrations listed in Table S4 (corresponding to the targeted molar ratios) and stirred for at least 1 h to ensure complete mixing; dopant solutions were prepared fresh on the day of use. All polymer and dopant solution preparation, including weighing reagents, was performed under an inert atmosphere (<5 ppm H_2_O and <10 ppm O_2_ during solution preparation; <5 ppm H_2_O and <10 ppm O_2_ during measurement).

### 4.3 Sample Preparation

All substrates (i.e., bare glass, and Si/SiO_2_ wafer) were ultrasonically cleaned in acetone, isopropyl alcohol, and deionized water, followed by drying in a vacuum oven for 3 h. Glass substrates for UV–vis measurements were cut into 1 cm squares. For TE measurements, four electrical contacts (Ti/Au, 5/50 nm) were deposited via sputtering through a shadow mask. Samples for GIWAXS measurement were prepared on a Si substrate. For AFM‐IR measurements, films were prepared on sputtered Au‐coated Si substrates. All substrates were treated with UV‐ozone for 15 min before use. P3HT films were spin‐coated onto preheated (120°C) patterned glass substrates (2000 rpm, 60 s) and subsequently annealed under N_2_ at 120°C for 10 min, followed by slow cooling down to room temperature. Films of PBTTT‐C_14_, PSBTBT, P(g2T‐TT), and PDPP‐3T were spin‐coated (2000 rpm, 60 s) onto cleaned pre‐patterned glass substrates and dried under N_2_ for 1–2 h prior to doping. Dopant solutions were prepared immediately before use. Films were doped using dopant‐specific solution‐processing protocols, including sequential immersion doping and spin‐cast doping (F_4_TCNQ:BCF); full processing conditions and post‐treatments are summarized in Section S3; Table S2a.

### 4.4 TE Measurements

The electrical conductivity was obtained using the equation *σ*  = 1/(*R*_s_ × *t*), where *R*_s_ is the sheet resistance measured by the four‐point probe method, and *t* is the thickness of the film. Resistance values were obtained using an Agilent source meter under a nitrogen atmosphere (<5 ppm H_2_O and <10 ppm O_2_). Thickness was measured using an AFM. The Seebeck coefficient was measured from a home‐made stage composed of two Peltier modules controlled with a power supply (Thurlby Thandar Instruments, PL320). Two K‐type thermocouples connected to the thermometer (Thermometer, TENMA 72–7712) were attached to the hot and cold sides of the films to detect the temperature. The generated thermovoltage was measured from the same leads used to measure temperature (to avoid the “cold finger” effect) using the Keithley 2000 multimeter. The Seebeck coefficient was extracted by the linear fitting of four pairs of Δ*T* and Δ*V* measurements. To account for the Seebeck coefficient of the probes, the setup was calibrated using a constantan reference sample. A correction factor of +7 µV/K was added to the relative Seebeck coefficient to obtain the absolute value. The temperature‐dependent electrical conductivity and Seebeck coefficient of the samples were measured by the commercial instrument (Linseis LSR‐3) under a He 0.5 bar overpressure atmosphere. A good agreement between the room‐temperature conductivity and Seebeck coefficient values measured with the home‐made setups and the commercial setup was found.

### 4.5 Spectroscopy

UV–vis–NIR spectra were collected on an Agilent Cary 6000i dual beam spectrometer, using a 2 nm slit width and 1 nm data interval. Substrate background spectra were collected separately. Spectra were recorded for both pristine and doped films using all dopants investigated in this study.

### 4.6 GIWAXS Characterization

Grazing‐incidence wide‐angle X‐ray scattering (GIWAXS) measurements were performed at BM26 (DUBBLE) beamline, European Synchrotron Radiation Facility (ESRF), Grenoble, France. Samples were probed with 12 keV X‐ray and a Pilatus 1 M detector was used for collecting GIWAXS data. The collected images were then processed using the GIXSGUI software [49]. The background was subtracted by fitting the curves to asymmetric least squares (ASLS), and peaks were fitted to pseudo‐Voigt functions. Peak widths and positions were used to calculate the π−π paracrystallinity, assuming the coherence length is dominated by paracrystalline disorder, as previously suggested by Rivnay et al. *g* = [Δ*q*/(2π×*q*)]^0.5^ where *q* and *Δq* are the position and FWHM of the peak, respectively [50].

### 4.7 Atomic Force Microscope‐Infrared Spectroscopy

Atomic force microscope‐infrared spectroscopy (AFM‐IR) measurements were carried out on a Bruker AFM‐IR platform with two lasers covering the mid‐IR region: laser A: 934–1666 cm^−1^, laser B: 1494–1934 cm^−1^. Samples were measured on Si substrates coated with a sputtered Au layer to provide a reflective surface for laser alignment. AFM‐IR spectra of pristine P3HT and dopant‐only (neat) TAB–2TFSI reference films were first collected to identify a TFSI^–^‐related vibrational marker band at 1350 cm^−1^ (see Section S6 for more details). The IR beam was aligned to the tip using the visible alignment laser, and AFM‐IR maps were subsequently recorded at 1350 cm^−^^1^. For mapping, AFM topography and phase images were acquired over the identical region of interest. All images were post‐processed using Gwyddion software.

## Author Contributions

**Juhyung Park**: conceptualization, investigation, funding acquisition, writing – original draft, methodology, validation, visualization, writing – review and editing, formal analysis, data curation. **Shubhradip Guchait**: conceptualization, investigation, writing – original draft, methodology, validation, visualization, writing – review and editing, formal analysis, data curation. **Tanmay Sinha**: methodology, writing – review and editing, funding acquisition, data curation, formal analysis. **Cynthia Verduyckt**: methodology, writing – review and editing. **Vijitha Ignatious**: methodology, writing – review and editing. **Tzu‐Yi Yu**: methodology, investigation, writing – review and editing. **Heyi Xia**: methodology, writing – review and editing. **Altynay Kaidarova**: methodology, writing – review and editing, funding acquisition. **Michael Ng**: methodology, writing – review and editing. **Siebe Detavernier**: methodology, writing – review and editing. **Martin Rosenthal**: methodology, writing – review and editing, formal analysis. **Maristella Alessio**: methodology, writing – review and editing. **Daniel Escudero**: funding acquisition, writing – review and editing, methodology. **Guy Koeckelberghs**: investigation, funding acquisition, writing – review and editing, methodology. **Francisco Molina‐lopez**: conceptualization, funding acquisition, writing – review and editing, validation, methodology, formal analysis, project administration, supervision, resources, visualization.

## Conflicts of Interest

The authors declare no conflicts of interest.

## Supporting information




**Supporting File**: advs77036‐sup‐0001‐SuppMat.docx.

## Data Availability

The data that support the findings of this study are openly available in the KU Leuven RDR repository (DOI: 10.48804/V5CNOO).
