# Supplementary material for: Dicationic Ion‐Pair Doping Enables Stable and High‐Performing Conducting Polymers for Organic Thermoelectrics
Source: Adv Sci (Weinh). 2026 Aug 15:e77036. Online ahead of print. doi: 10.1002/advs.77036 (PMC13477255; doi:10.1002/advs.77036)
Supplement: Supplementary file 1 [file ADVS-9999-e77036-s001.docx]

Supporting Information

**Dicationic Ion-Pair Doping Enables Stable and High-Performing Conducting Polymers for Organic Thermoelectrics**

Juhyung Park^†^, Shubhradip Guchait^†^, Tanmay Sinha, Cynthia Verduyckt, Vijitha Ignatious, Tzu-Yi Yu, Heyi Xia, Altynay Kaidarova, Michael Ng, Siebe Detavernier, Martin Rosenthal, Maristella Alessio, Daniel Escudero, Guy Koeckelberghs, Francisco Molina-Lopez*

^†^ First authors

Section S1. Synthesis of P3HT and TAB-2TFSI

**Synthesis of P3HT**

2-bromo-3-hexylthiophene (0.035 mol; 8.652 g; 1 eq) was brought into a flask under inert atmosphere and diluted with dry THF (250 mL; 0.14 M). While stirring at room temperature, 2,2,6,6-tetramethylpiperidinylmagnesium chloride lithium chloride complex solution (TMPMgCl·LiCl) (0.035 mol; 35.0 mL; 1.0 M) was added to the monomer solution. After reacting for 3 h, the monomer solution was added to a solution of Ni(dppp)Cl_2_ (0.350 mmol; 0.190 g) in 100 mL dry THF while stirring at room temperature. The polymerization mixture was reacted for 1 h before terminating with acidified THF. The resulting polymer was then precipitated into methanol and filtered over a Soxhlet thimble. The polymer was purified by Soxhlet extraction with methanol and chloroform after which the polymer was again precipitated into methanol. P3HT was obtained as a purple solid (*m* = 3.500 g; yield = 60 %). The polymer was characterized via size exclusion chromatography to determine its number-average molar mass (*M*_n_) and dispersity (*Ð*) (*M*_n_ = 16.3 kg mol^−1^ and *Ð* = 1.36).

Scheme S1. Synthesis of TAB–2TFSI

Synthesis of TAB–2TFSI

TAB–2TFSI was synthesized following a previously reported procedure.^[1]^ As shown in Scheme S1, two consecutive reaction steps were performed. First, 4,4’-[N,N,N’,N’-tetrakis(4-bromophenyl)-diamino]biphenyl (TAB) was synthesized by adding NBS (0.120 mol; 21.358 g; 4.00 eq.) to a solution of N,N,N’,N’-tetraphenylbenzidine (0.030 mol; 14.659 g; 1.00 eq.) in DMF (210 mL, 0.14 M) at 0 °C under a nitrogen atmosphere and shielded from light. The mixture was stirred overnight and then precipitated in methanol. The precipitate was collected by vacuum filtration to afford a white solid (*m* = 23.458 g; yield = 97 %). ^1^H-NMR (600 MHz, CDCl3) *δ* (ppm) 7.45 (4H, J = 8.04 Hz), 7.36 (8H, J = 8.16 Hz), 7.09 (4H, J = 8.04 Hz), 6.97 (8H, J = 8.16 Hz).

To obtain TAB-2TFSI, a solution of TAB (0.010 mol; 8.042 g; 1.00 eq.) and AgTFSI (0.030 mol; 11.640 g; 3.00 eq.) in diethyl ether (800 mL) was cooled to 0 °C. A solution of iodine (0.030 mol; 7.614 g; 3.00 eq.) in diethyl ether (100 mL) was added dropwise to the mixture. After the addition of iodine, the mixture was stirred for 30 min. The brown precipitate, containing AgI and the product was collected by filtration. This precipitate was dissolved in DCM and filtered over celite to remove AgI. The solvent was removed under reduced pressure and the product was redissolved in DCM. This solution was precipitated in diethyl ether and filtered using a Büchner filter, to afford wine red crystals (*m* = 6.213 g; yield = 46 %). ^19^F-NMR (CD_2_Cl_2_): −79.3 ppm (s).

Characterization

*Size exclusion chromatography (SEC)*: SEC measurements were performed on a Shimadzu LC20 apparatus (PLgel 5 μm MIXED-E column, LC-20AT pump, CBM-20A controller, SPD20A and RID-20A detectors) using THF as the eluent for determining the molar masses of the polymer. The SEC apparatus was calibrated with polystyrene standards (Polymer Laboratories) in THF. Before the measurement, the sample was fully dissolved/diluted in THF (concentration ≈ 1 mg mL^−1^) and filtered (pore size = 0.2 μm).

*NMR spectroscopy*: 1H-NMR and 19F-NMR spectra were recorded on a Bruker Avance III HD 400 and Bruker Avance II+ 600 spectrometers.

Section S2. Thermodynamic evaluation of the reduction of TAB^2+^

Using density functional theory (DFT), we estimated the cell potentials for the half-cell reactions associated with the reduction of TAB^2+^ to TAB^0^ with respect to the vacuum. The following reactions were considered: 1) TAB^2+^ → TAB^0^, 2) TAB^2+^ → TAB^+^, and 3) TAB^+^ → TAB^0^. All calculations were performed in vacuum using the ωB97XD functional in combination with the def-TZVP basis set. The cell potentials were obtained from the reaction Gibbs free energies using the relation Δ*G* = −*nFE*_cell_, where *n* is the number of electrons transferred and *F* is the Faraday constant.

For the direct two-electron reduction of TAB^2+^ to TAB^0^ in a single step, the calculated cell potential is 8.07 V. In contrast, for a stepwise mechanism involving sequential one-electron reductions, the first reduction step (TAB^2+^ → TAB⁺) yields a cell potential of 9.49 V, followed by a second reduction step (TAB⁺ → TAB^0^) that also exhibits a positive potential of 6.65 V. These positive values indicate that the reduction toward TAB^0^ is thermodynamically favorable. Moreover, the higher potential for the first one-electron reduction suggests that the initial attachment of a single electron to TAB^2+^ is more favorable than a concerted two-electron process. Nevertheless, because the second reduction step also has a positive potential, the reaction pathway is still expected to proceed toward the fully reduced TAB^0^ species.

Table S1. Thermodynamic parameters for the reduction of TAB^2+^. Gibbs free energies and cell potentials were computed using the ωB97XD functional and def-TZVP basis set.

| Reaction steps | Gibbs free energies Δ*G* (eV) | Cell potentials *E*_cell_ (V) |
| --- | --- | --- |
| TAB^2+^ → TAB^0^ | −16.14 | 8.07 |
| TAB^2+^ → TAB^+^ | −9.49 | 9.49 |
| TAB^+^ → TAB^0^ | −6.65 | 6.65 |

Section S3. Film doping protocols and optical estimation of the doping state

All doping solutions were prepared immediately prior to use due to the limited stability of several dopants in the presence of trace moisture. Unless otherwise noted, dopant solution preparation and film doping were carried out in a nitrogen-filled glovebox. An exception was the F_4_TCNQ:BCF protocol, which was intentionally performed in air to enable the Brønsted acid–assisted doping mechanism.^[2]^ Films were doped either by immersion in dopant solutions or, for the F4TCNQ:BCF system, by a spin-cast doping protocol.^[3]^

Immersion doping and post-treatments

For immersion doping, polymer films on patterned substrates were immersed in freshly prepared dopant solutions under the processing temperature and time specified in Table S2a to maximize conductivity (see Table S4). For mixed dopant systems (FeCl_3_/Li–TFSI and F_4_TCNQ/Li–TFSI), both components were co-dissolved in a single solution at the concentrations given in Table S2a, and doping was performed in a single immersion step. For protocols conducted at elevated temperature, the dopant solution was pre-equilibrated to the target temperature prior to immersion. After immersion, films were removed from the solution, gently drained, and transferred directly to post-treatment or measurement without rinsing, as rinsing led to a noticeable decrease in conductivity in our tests. For TAB–2TFSI, FeCl_3_/Li–TFSI, FeCl_3_, F_4_TCNQ, and F_4_TCNQ/Li–TFSI, the immersion was performed at 60 °C for 10 min (Table S2a), followed by a post-annealing step at 60 °C for 10 min after removal from solution. In contrast, Magic Blue–doped films were immersion-doped at room temperature for 1 min, then removed from the dopant solution and gently dried (e.g., N_2_ blowing) prior to measurement without post-annealing, as thermal treatment reduced conductivity.

Spin-cast protocol for F_4_TCNQ:BCF

For the F_4_TCNQ:BCF system, we adopted a sequential spin-cast doping protocol adapted from a previously reported method.^[3]^ Briefly, a mixed dopant solution of F_4_TCNQ and BCF in ACN:EA (95:5, v/v) was spin-cast onto pre-formed P3HT films under ambient atmosphere (3000 rpm, 30 s; Table S2a). Unless otherwise specified, a F_4_TCNQ:BCF molar ratio of 1:5 was employed (corresponding to 1:10 wt/wt in the reference protocol). After deposition, the films were annealed at 120 °C for 10 s in air to promote dopant incorporation and equilibration. To remove excess dopant/acid residues, a solvent-extraction step was subsequently performed by spin-casting neat acetonitrile (100 µL, 3000 rpm) onto the doped films. Throughout this work, the “processing time” refers to the immersion time for immersion-doped samples and to the spin-coating duration for the spin-cast F_4_TCNQ:BCF protocol. A concise summary of the representative processing conditions used to obtain the maximum room-temperature conductivity for each dopant/counterion strategy is provided in Table S2a. Concentration-dependent thermoelectric datasets are presented later in the Supporting Information.

Table S2a. Processing conditions for each dopant system that yielded the maximum electrical conductivity. Processing conditions of P3HT films shown in Fig. 1d (see Table S4 for dopant concentration optimization). For mixed systems, concentrations are reported as A/B in the order shown in the dopant column (e.g., FeCl_3_/Li-TFSI, F_4_TCNQ/Li-TFSI and F_4_TCNQ:BCF). All solution preparation and doping processes were performed in a nitrogen-filled glovebox, except for the F_4_TCNQ:BCF, which was processed in air. For immersion doping, the processing time corresponds to the immersion time. For the F_4_TCNQ:BCF (spin-cast), it corresponds to the spin-coating duration (3000 rpm).

| Dopant | Solvent | Concentration (mM) | Processing  (°C, min) | Annealing  (°C, min) | Method |
| --- | --- | --- | --- | --- | --- |
| TAB–2TFSI | ACN | 1.5 | 60, 10 | 60, 10 | Immersion |
| Magic Blue | ACN | 0.5 | RT, 1 | — | Immersion |
| FeCl_3_/Li-TFSI | ACN | 1.5 / 7.5 | 60, 10 | 60, 10 | Immersion |
| FeCl_3_ | ACN | 2 | 60, 10 | 60, 10 | Immersion |
| F_4_TCNQ/Li-TFSI | ACN | 5 / 25 | 60, 10 | 60, 10 | Immersion |
| F_4_TCNQ:BCF | ACN: EA  (95:5) | 32.5 / 175 | RT, 0.5 | 120, 0.15 | Spin-cast |
| F_4_TCNQ | ACN | 5 | 60, 10 | 60, 10 | Immersion |

Abbreviations: ACN, acetonitrile; EA, ethyl acetate; RT, room temperature.

Optical estimation of carrier density and mobility

We estimated the charge carrier density of the doped films from their UV–Vis–NIR spectra, following the spectroelectrochemical calibration approach reported by Untilova et al.^[4]^ In that work, the attenuation coefficient of the P2 polaron band was determined by combining chronoamperometry and spectroelectrochemistry. The carrier density was estimated from the Beer–Lambert relation *N*_v,opt_ = Δ*A*_800_·*N*_A_/(*ε*_P2_·*d*), where Δ*A*_800_ is the difference in absorbance at 800 nm between the doped and pristine films, *N*_A_ is Avogadro's constant, *d* is the film thickness (ca. 50 ± 5 nm for our films), and *ε*_P2_ = 4.1 × 10^3^ m^2^ mol^−1^ at 800 nm is a fitting coefficient extracted experimentally by Untilova et al.^[4]^ for P3HT. Since the different dopants used in our study do not alter the absorption pattern of P3HT significantly, we assumed the same *ε*_P2_ coefficient applies to all the P3HT-dopant systems tested in our study. The oxidation level was calculated by dividing *N*_v,opt_ by the P3HT thiophene-ring density (4.0 × 10^27^ m^−3^), and a mobility estimate was calculated from the measured conductivity using the Drude model *μ*_opt_ = *σ*/(*q*·*N*_v,opt_), where *σ* is the measured conductivity and *q* is the elementary charge. The resulting values are summarized in Table S2b.

Table S2b. Optical estimation of charge carrier density, oxidation level, and mobility in doped P3HT films. *N*_v,opt_, oxidation level, and *μ*_opt_ were estimated from the P2 polaron absorption at 800 nm as described above.

| Dopant | Δ*A*_800_ | *N*_v,opt_ (10^26^ m^−3^) | Oxidation level (%) | *μ*_opt_ (cm^2^ V^−1^ s^−1^) |
| --- | --- | --- | --- | --- |
| TAB–2TFSI | 0.199 | 5.3 | 13.3 | 3.4 |
| Magic Blue | 0.151 | 4.9 | 12.4 | 3.1 |
| FeCl_3_/Li-TFSI | 0.162 | 5.1 | 12.6 | 3.0 |
| FeCl_3_ | 0.153 | 4.8 | 12.0 | 2.8 |
| F_4_TCNQ:BCF | 0.104 | 3.0 | 7.6 | 1.9 |

Section S4. GIWAXS linecut analysis and fitting methodology

Linecut processing, peak fitting, and summary of fitting curves

To rigorously quantify the structural parameters of pristine and doped P3HT films, 1D linecuts were extracted from the 2D GIWAXS patterns along both the in-plane *q*_ip_ and out-of-plane *q*_oop_ directions. The raw scattering data were processed through a systematic numerical procedure designed to isolate crystalline reflections from extraneous background signals. Initially, the contribution from the Si substrate was subtracted from the raw linecuts to isolate the scattering intensity originating solely from the polymer thin film. Subsequently, baseline correction was performed using the Asymmetric Least Squares (AsLS) algorithm to effectively estimate the broad background signal, primarily originating from the amorphous halo of the polymer backbones and diffuse scattering. This approach ensured a physically consistent baseline that followed the underlying amorphous profile without distorting the shape or intensity of the crystalline reflections. Finally, the baseline-subtracted profiles were deconvoluted using multiple Pseudo-Voigt functions, which effectively modeled the peak shapes by combining Gaussian and Lorentzian broadening components. As illustrated in Figure S1 using the pristine P3HT film as a representative example, this fitting protocol enabled the precise resolution of overlapping peaks. Specifically, the (002) backbone reflection (≈1.2–1.35 Å^−1^) and the (020) π–π stacking reflection (≈1.6–1.7 Å^−1^) were successfully resolved in the in-plane (IP) direction (Figure S1a), while the (*h*00) lamellar reflections were identified in the out-of-plane (OOP) direction (Figure S1b). For comparative clarity, the total fitting curves for all doping conditions are summarized in the waterfall plots (Figure S2a and S2b). The structural parameters presented in Figure 2b and Table S3 (e.g., *d*-spacing and *g*-factor) were calculated by averaging the results from at least three independent samples (*n* ≥ 3), measured across different experimental batches to ensure statistical reliability and reproducibility. A representative example of a broadened/ill-defined case (F_4_TCNQ:BCF) and the corresponding exclusion rationale are provided below (Section S5 and Figure S3).

Figure S1. Representative deconvolution of GIWAXS 1D linecuts. Representative peak deconvolution and fitting procedure for a pristine P3HT film along the (a) in-plane *q*_ip_ and (b) out-of-plane *q*_oop_ directions. The blue circles denote experimental data after substrate subtraction, and the dashed lines indicate the baseline. Individual crystalline reflections (hatched areas) were resolved using multiple Pseudo-Voigt functions, and the black solid lines represent the total fit.

Figure S2. Comparative 1D GIWAXS profiles for various doping conditions. Comparative 1D GIWAXS linecuts of pristine and doped P3HT films extracted along the (a) in-plane *q*_ip_ and (b) out-of-plane *q*_oop_ directions. The profiles are displayed as waterfall plots for clarity, where each curve represents the total Pseudo-Voigt fit after substrate subtraction and baseline correction. (a) In-plane profiles highlighting the (002) backbone and (020) π–π stacking reflections. The vertical dashed lines serve as guides to emphasize the peak shifts and changes in crystallinity upon doping. (b) Out-of-plane profiles showing the evolution of (*h*00) lamellar reflections. The intensities are plotted on a linear scale to accurately represent the relative differences in scattering strength and peak widths (FWHM) among the samples.

Table S3. GIWAXS structural parameters for pristine and doped P3HT films. Parameters were derived from pseudo-Voigt fits of baseline-corrected 1D linecuts along the out-of-plane (OOP) and in-plane (IP) directions (see Section S4, Figure S1 and S2). d-spacings were calculated as *d* = 2π/*q*. The g-factor was extracted from the IP (020) (π–π stacking) reflection following the same procedure described in Section S4 and using the equation $\sqrt{\frac{\boldsymbol{FWHM}}{\boldsymbol{2}\boldsymbol{\pi q}}}$ ∙100. All values are reported as mean ± standard deviation (n ≥ 3).

| Sample | Orientation & reflection | *q* (Å^−1^) | *d*-spacing (Å) | FWHM (10^−3^ Å^−1^) | *g*-factor (%) |
| --- | --- | --- | --- | --- | --- |
| Pristine | OOP (100) | 0.40 ± 0.02 | 15.8 ± 0.6 | 44 ± 7 | — |
|  | IP (020) | 1.66 ± 0.03 | 3.79 ± 0.01 | 90 ± 5 | 9.3 ± 0.3 |
| TAB–2TFSI | OOP (100) | 0.36 ± 0.01 | 17.4 ± 0.4 | 41 ± 7 | — |
|  | IP (020) | 1.71 ± 0.06 | 3.67 ± 0.01 | 88.0 ± 0.3 | 9.1 ± 0.2 |
| FeCl_3_/Li-TFSI | OOP (100) | 0.36 ± 0.01 | 17.3 ± 0.5 | 40 ± 1 | — |
|  | IP (020) | 1.71 ± 0.01 | 3.68 ± 0.02 | 100 ± 2 | 9.6 ± 0.1 |
| F_4_TCNQ/Li-TFSI | OOP (100) | 0.36 ± 0.01 | 17.6 ± 0.3 | 47 ± 6 | — |
|  | IP (020) | 1.68 ± 0.01 | 3.74 ± 0.02 | 93 ± 4 | 9.4 ± 0.2 |
| Magic Blue | OOP (100) | 0.37 ± 0.01 | 17.1 ± 0.5 | 39 ± 2 | — |
|  | IP (020) | 1.71 ± 0.02 | 3.67 ± 0.01 | 113 ± 5 | 10.3 ± 0.2 |
| FeCl_3_ | OOP (100) | 0.37 ± 0.01 | 16.9 ± 0.2 | 40 ± 1 | — |
|  | IP (020) | 1.71 ± 0.02 | 3.67 ± 0.01 | 96 ± 4 | 9.4 ± 0.2 |
| F_4_TCNQ | OOP (100) | 0.37 ± 0.01 | 17.0 ± 0.2 | 41 ± 1 | — |
|  | IP (020) | 1.67 ± 0.01 | 3.76 ± 0.01 | 93 ± 3 | 9.4 ± 0.1 |

Section S5. GIWAXS of F_4_TCNQ:BCF-doped P3HT films

Figure S3 presents the 2D GIWAXS pattern and 1D linecuts of F_4_TCNQ:BCF-doped P3HT films. Unlike the optimized TAB–2TFSI system, the F_4_TCNQ:BCF-doped samples exhibit extreme peak broadening and a loss of distinct crystalline features, as seen in the 2D map (Figure S3a) and the ill-defined reflections in both in-plane (Figure S3b) and out-of-plane (Figure S3c) directions. Because distinct crystalline reflections could not be reliably assigned and quantitative peak fitting did not yield robust d-spacing and paracrystallinity parameters, the F_4_TCNQ:BCF datasets were not included in the quantitative summary.

Figure S3. Representative GIWAXS analysis for F_4_TCNQ:BCF-doped P3HT films. (a) 2D GIWAXS pattern of an F_4_TCNQ:BCF-doped P3HT thin film. (b) 1D in-plane *q*_ip_ and (c) out-of-plane *q*_oop_ linecuts extracted from the 2D pattern. In contrast to the TAB–2TFSI-doped films, the F_4_TCNQ:BCF-doped samples exhibit significantly broadened crystalline reflections in both directions. Specifically, the broadened (*h*00) lamellar reflections in (c) and the increased width of the (020) π–π stacking reflection in (b) indicate a higher degree of structural disorder and compromised long-range crystalline order.

Section S6. AFM-IR Methodology

**AFM-IR mapping and signal definition**

Atomic force microscopy–infrared spectroscopy (AFM-IR) mapping was used to probe the nanoscale spatial distribution of TFSI⁻ counterions in doped P3HT films. AFM-IR measurements were performed on a Bruker AFM-IR platform equipped with a dual-laser configuration (laser A: 934–1666 cm^−1^, laser B: 1494–1934 cm^−1^), enabling coverage of the mid-IR spectral range relevant to this study. In AFM-IR, local infrared absorption is detected through the photothermal expansion of the sample under a pulsed, tunable IR laser while the AFM tip is in contact with the surface, enabling chemical imaging beyond the optical diffraction limit. For each sample, AFM topography (and phase, when available) was acquired to confirm stable tip–sample contact and to assess whether the chemical contrast correlates with morphological features. Chemical maps were then collected by recording the AFM-IR response at a fixed wavenumber across a defined scan area, generating a 2D intensity map that reflects the relative abundance of the targeted vibrational marker. The AFM-IR signal is reported as the cantilever oscillation amplitude (mV), corresponding to the photodetector response to the IR-induced tip/sample oscillation; accordingly, the spectra and maps are presented as relative signal intensities rather than absolute absorbance.

Figure S4. Reference AFM-IR response spectra used to select the TFSI⁻ marker band. Overlaid AFM-IR amplitude spectra of drop-cast TAB–2TFSI layer (red) and pristine P3HT film (black) in the 900–1700 cm⁻^1^ range. The inset (1300–1400 cm⁻^1^) highlights a pronounced band near ~1350 cm⁻^1^ for TAB–2TFSI with negligible signal for pristine P3HT.

Preparation of AFM-IR reference samples

TAB–2TFSI was dissolved in acetonitrile (1.5 mM). A 10 µL aliquot was drop-cast onto a Si substrate and dried in a glovebox at room temperature for 24 h. The resulting thin coating was used only to acquire the reference spectrum in Figure S4. The pristine P3HT film was prepared identically to the procedure described in the Methods section of the main text.

Selection of the TFSI marker band (1350 cm^−1^)

To selectively visualize TFSI⁻, we monitored the IR response at 1350 cm^−1^. This wavenumber was chosen as a practical and robust TFSI-related marker band because it provides clear spectral contrast between TFSI-containing species and pristine P3HT within the same measurement window (Figure S4).^[5]^ As shown in Figure S4, the drop-cast TAB–2TFSI film reference exhibits a discernible AFM-IR response feature around 1350 cm^−1^, whereas pristine P3HT shows negligible response in this region under identical conditions, enabling reliable visualization of TFSI⁻ incorporation with minimal interference from polymer vibrations. The 1350 cm^−1^ band is expected to originate primarily from SO_2_ asymmetric stretching of TFSI⁻, while S–N and C–F stretching vibrations can also contribute in this spectral region; therefore, we refer to this feature conservatively as a TFSI-related band rather than assigning a single vibrational mode.

Section S7. Temperature-dependent conductivity and Seebeck measurements

**Measurement setup and protocol (LSR-3)**

The temperature-dependent electrical conductivity, *σ*(*T*), and Seebeck coefficient, *α*(*T*), were measured using a commercial thermoelectric measurement system (Linseis LSR-3) under a helium atmosphere with 0.5 bar overpressure. For the representative measurements reported here, the temperature was swept over ~160–350 K using a nominal setpoint increment of ~5–10 K. Electrical conductivity was obtained in a four-probe configuration on thin-film samples with a probe spacing of 6 mm. The reported σ values were calculated from the measured resistance using the sample geometry between the inner electrodes (*L*_in_ = 3.6 mm, *W* = 17 mm) together with the film thickness. At each temperature setpoint (after reaching steady state), the Seebeck coefficient was determined from the slope of the thermovoltage as a function of an applied temperature difference, Δ*T*. During the measurement, the applied temperature difference was typically ~2.6–4.0 K. At each temperature setpoint, three successive data points were recorded and averaged; the uncertainties shown in *σ*(*T*) and *α*(*T*) represent the standard deviation of multiple measurements (*n* = 3) unless noted otherwise.

Arrhenius analysis and temperature-dependent transport behaviors

Figure S5a presents an Arrhenius-type representation of the conductivity, where lnσ is plotted as a function of inverse temperature and fit linearly to extract an apparent activation energy for transport, E_a_. Assuming thermally activated behavior, the conductivity can be expressed as

$$\begin{aligned} \sigma\left( T \right)=\sigma_{0}\exp\left( -\frac{E_{a}}{k_{B}T} \right),\#(S1) \end{aligned}$$

which gives

$$\begin{aligned} \ln\sigma=\ln\sigma_{0}-\frac{E_{a}}{k_{B}}\left( \frac{1}{T} \right).\#(S2) \end{aligned}$$

Accordingly, *E_a_* was obtained from the slope of the linear fit, slope = −*E*_a_/*k*_B_ (Figure S5a), where *k*_B_ is a Boltzmann constant. The temperature-dependent Seebeck coefficient *α*(*T*) was measured over the same temperature window (Figure S5b), enabling subsequent analysis using the Kang–Snyder transport model analysis discussed below.

Figure S5. Temperature-dependent transport properties measured by LSR-3. (a) Arrhenius-type plots of electrical conductivity, ln*σ*, as a function of 1000/*T* for representative doped P3HT films (TAB–2TFSI, Magic Blue, FeCl_3_/Li–TFSI, and FeCl_3_). Dashed lines show linear fits used to extract an apparent activation energy for charge transport. (b) Temperature-dependent Seebeck coefficient, *α*(*T*), measured over the same temperature window. Error bars represent the standard deviation from three successive measurements at each temperature setpoint (*n* = 3).

Section S8. Room-temperature thermoelectric datasets and measurement protocol

To establish Seebeck–conductivity (*α*–*σ*) datasets at room temperature for the transport analysis in the main text, we measured the electrical conductivity (*σ*) and Seebeck coefficient (*α*) as a function of dopant concentration using a custom thermoelectric measurement setup. For each dopant/counterion strategy, films were prepared under consistent processing conditions (Section S3). The device geometry and measurement procedure follow recent recommendations for consensus reporting of organic thermoelectric measurements.^[6]^

Unless noted otherwise, measurements were performed inside a nitrogen-filled glovebox using patterned Ti (5 nm)/Au (50 nm) electrodes on a glass substrate uniformly covered by the polymer film (Figure S6a). Electrical conductivity was measured in a four-probe configuration by sourcing current through the outer current-carrying electrodes (*I*_σ_) and recording the voltage drop across the inner voltage probes (*V*_σ_). For conductivity calculations, the channel geometry was defined by the inner-probe spacing and film width (*L*_in_ = 4 mm, *W* = 14 mm), together with the measured film thickness. The outer electrode spacing (*L*_out_ = 11 mm) and electrode length (*L*_E_ = 1.5 mm) are indicated in Figure S6a.

Figure S6. Device geometry for room-temperature thermoelectric measurements. (a) Schematic of a polymer film on patterned Ti (5 nm)/Au (50 nm) electrodes on a glass substrate. Electrical conductivity was measured in a four-probe configuration by sourcing current through the outer electrodes *I_σ_* and recording the voltage drop across the inner probes *V_σ_*. The Seebeck coefficient was obtained from the thermovoltage (*V*_TH_) between the same inner probes under a lateral temperature gradient, with Δ*T* = *T*_hot_ – *T*_cold_ monitored adjacent to the probes. (b) Representative Δ*T*–Δ*V* data acquired near room temperature (mean temperature ≈ 23 °C) and the corresponding linear fit used to extract *α* from the slope (*α* = d*V*/d*T*).

On the same device, the Seebeck coefficient was measured by applying a lateral temperature difference using a heater–cooler pair (Peltier modules) and recording the thermovoltage (*V*_TH_) between the same inner voltage probes used for *σ*. The temperature difference was defined as, Δ*T* = *T*_hot_ – *T*_cold_, where *T*_hot_ and *T*_cold_ were monitored adjacent to the inner probes (Figure S6a), minimizing uncertainty associated with temperature drops outside the active channel. To keep the average sample temperature close to room temperature (≈23 °C) while varying Δ*T*, *T*_hot_ and *T*_cold_ were adjusted symmetrically around the target mean temperature (e.g., *T*_cold_/*T*_hot_ = 22.0/24.0 °C, 21.5/24.5 °C, 21.0/25.0 °C and 20.5/25.5 °C). The Seebeck coefficient was then extracted from the slope of a linear fit of Δ*V* versus Δ*T* (*α* = d*V*/d*T*), as illustrated by a representative Δ*V*–Δ*T* dataset in Figure S6b. Using multiple Δ*T*–Δ*V* pairs and a linear fit reduces the influence of parasitic voltage offsets and improves the robustness of *α* extraction. The power factor was calculated as *PF* = *α*^2^*σ*. For each dopant concentration, *σ* and *α* were obtained from the same device and reported as paired values. Unless noted otherwise, the reported values and error bars represent the standard deviation from at least three independently prepared devices (*n* ≥ 3) for each condition. The resulting concentration-dependent *σ*, *α*, and *PF* datasets are summarized in Figure S7 and Table S4.

Figure S7. Thermoelectric properties of doped P3HT films. (a–c) Electrical conductivity *σ*, Seebeck coefficient *α*, and power factor *PF* as a function of dopant concentration for TAB–2TFSI, Magic Blue, FeCl_3_/Li-TFSI, and FeCl_3_ systems. For the FeCl_3_/Li-TFSI system, the Li-TFSI concentration was fixed at fivefold excess relative to FeCl_3_, i.e., [Li-TFSI] = 5[FeCl_3_] (d–f) *σ*, *α*, and *PF* for the F_4_TCNQ:BCF system plotted versus F_4_TCNQ concentration, with BCF maintained at fivefold excess, [BCF] = 5[F_4_TCNQ]. PF was calculated as *PF* = *α*^2^*σ*.

**Table S4. Thermoelectric properties of doped P3HT films**

Electrical conductivity *σ*, Seebeck coefficient *α*, and power factor *PF* of doped P3HT films as a function of dopant concentration for each doping system. Values are reported as mean ± standard deviation from three independently prepared samples. For mixed systems, concentrations are reported as A/B in the order shown in the dopant column.

| Doping system | Concentration (mM) | *σ* (S cm^−1^) | *α* (µV K^−1^) | *PF* (µW m^−1^ K^−2^) |
| --- | --- | --- | --- | --- |
| TAB–2TFSI  (immersion) | 0.1 | 24 ± 2 | 79 ± 3 | 15 ± 2 |
|  | 0.2 | 98 ± 9 | 57 ± 3 | 32 ± 5 |
|  | 0.5 | 162 ± 15 | 50 ± 3 | 40 ± 6 |
|  | 1 | 251 ± 20 | 32 ± 2 | 25 ± 4 |
|  | 1.5 | 290 ± 30 | 28 ± 2 | 22 ± 3 |
|  | 2 | 290 ± 30 | 27 ± 2 | 20 ± 3 |
| Magic Blue  (immersion) | 0.1 | 185 ± 17 | 41 ± 2 | 31 ± 4 |
|  | 0.2 | 220 ± 20 | 31 ± 1 | 22 ± 2 |
|  | 0.3 | 240 ± 20 | 27 ± 1 | 17 ± 2 |
|  | 0.4 | 240 ± 20 | 28 ± 1 | 19 ± 2 |
|  | 0.5 | 250 ± 20 | 23 ± 1 | 13 ± 1 |
|  | 0.6 | 240 ± 20 | 28 ± 1 | 19 ± 2 |
| FeCl_3_/Li-TFSI  (immersion) | 0.5 / 2.5 | 48 ± 4 | 72 ± 2 | 25 ± 2 |
|  | 1 / 5 | 227 ± 18 | 31 ± 2 | 22 ± 3 |
|  | 1.5 / 7.5 | 242 ± 19 | 27 ± 1 | 18 ± 2 |
| FeCl_3_  (immersion) | 0.5 | 4.1 ± 0.3 | 143 ± 2 | 9 ± 1 |
|  | 0.6 | 15 ± 1 | 87 ± 4 | 11 ± 1 |
|  | 0.8 | 58 ± 4 | 64 ± 1 | 24 ± 2 |
|  | 1 | 88 ± 6 | 58 ± 2 | 30 ± 3 |
|  | 1.5 | 167 ± 12 | 42 ± 1 | 30 ± 3 |
|  | 2 | 215 ± 15 | 31 ± 1 | 20 ± 1 |
| F_4_TCNQ:BCF  (spin-cast) | 6.5 / 35 | 49 ± 4 | 43 ± 1 | 9 ± 1 |
|  | 9.75 / 52.5 | 73 ± 6 | 49 ± 1 | 17 ± 2 |
|  | 16.25 / 87.5 | 46 ± 4 | 47 ± 2 | 10 ± 1 |
|  | 19.5 / 105 | 83 ± 7 | 51 ± 2 | 22 ± 3 |
|  | 26 / 140 | 92 ± 8 | 40 ± 1 | 15 ± 1 |
|  | 32.5 / 175 | 86 ± 9 | 36 ± 2 | 11 ± 2 |

Section S9. Kang–Snyder transport model analysis

To analyze the Seebeck–conductivity trends of doped P3HT films, we employed the Kang–Snyder (K–S) transport framework,^[7]^ which parameterizes the α–σ relationship using the reduced chemical potential η and a material-dependent transport coefficient σ_E0_. In the K–S description, α and σ are expressed as functions of η and the transport parameter s through complete Fermi–Dirac integrals F_j_(η):

$$\begin{aligned} \alpha\left( \eta\right)=\frac{k_{B}}{e}\left[ \frac{\left( s+2)F_{s+1}(\eta\right)}{\left( s+1)F_{s}(\eta\right)} - \eta\right],\#(S3) \end{aligned}$$

$$\begin{aligned} \sigma\left( \eta\right)=\sigma_{E0}\text{ }F_{s}\left( \eta\right),\#(S4) \end{aligned}$$

$$\begin{aligned} F_{j}\left( \eta\right)=\int_{0}^{\infty} \frac{\varepsilon^{j}}{1+\exp\left( \varepsilon-\eta\right)}\text{ }d\varepsilon.\#(S5)\# \end{aligned}$$

In this framework, *η* = (*E*_F_ − *E*_t_)/*k*_B_*T* is the reduced chemical potential that captures how the Fermi level is positioned relative to the transport level, where *E*_F_ and *E*_t_ are the chemical potential and transport edge, respectively. The transport coefficient *σ*_E0_ is a material-dependent transport coefficient that sets the overall conductivity scale for a given transport parameter *s* (*i.e.,* it reflects the effective mobility/connectivity of the conducting network, independent of the instantaneous Fermi-level position encoded in *η*). The exponent *s* governs the energy dependence of transport and thus determines the shape of the *α*–*σ* relationship through the Fermi–Dirac integral.

Extraction of *σ*_E0_ at room temperature (*σ*_E0_(RT))

Room-temperature *α*–*σ* pairs were analyzed within the K–S framework to extract *η*(RT) and *σ*_E0_(RT). Specifically, *η*(RT) was obtained by solving Equation S3 for *η* using the measured *α*(RT). The corresponding *σ*_E0_(RT) was then calculated from Equation S4. The resulting *σ*_E0_(RT) values were used to construct the K–S curves and compare dopant/counterion strategies at room temperature (main text, Figure 4c). As shown in Figure 4c, the highly doped samples are well captured by the *s* = 1 K–S curves, whereas less-doped samples progressively deviate toward a weaker *σ*-dependence of *α* (approaching an *α*–*σ* power-law trend with an exponent of –1/4), which is often discussed in polymer thermoelectrics in terms of increasing carrier localization at lower doping levels.^[8]^

Temperature-dependent *σ*_E0_(*T*) and extraction of the transport barrier *W_γ_*

To extract the transport-barrier parameter, σ_E0_(T) was obtained from temperature-dependent matched α(T)–σ(T) pairs. For each dopant/counterion series, we selected the highest-conductivity (most heavily doped) sample and determined η(T) at each temperature by solving Equation S3 using the measured α(T). σ_E0_(T) was then calculated from

$$\begin{aligned} \sigma_{E0}\left( T \right)=\frac{\sigma\left( T \right)}{F_{s}\left( \eta\left( T \right) \right)}.\#(S6) \end{aligned}$$

In many thin-film studies, temperature-dependent *σ*_E0_(*T*) is instead estimated by first determining *η* at a single temperature (typically room temperature) and then assuming a temperature-independent energy offset Δ*E* = *E*_F_ –*E*_t_, such that *η*(*T*) = Δ*E*/*k*_B_*T* is imposed to follow a 1/*T* dependence. This approximation can become unreliable if the effective energetic landscape evolves with temperature or doping level (e.g., changes in transport level alignment, energetic disorder, or carrier localization). Here, because we directly measured matched *α*(*T*) and *σ*(*T*) over the full temperature range, we determined *η*(*T*) at each temperature from *α*(*T*) and thus obtained *σ*_E0_(*T*) without imposing a fixed Δ*E* assumption, enabling a more experimentally grounded extraction of the transport-barrier parameter. The transport-barrier parameter *W*_γ_ (*γ* = 1/2) was extracted from the linear relation in ln*σ*_E0_ vs *T*^−1/2^ plots (Figure S8):

$$\begin{aligned} \ln\sigma_{E0}\left( T \right)=\ln\sigma_{0}-\left( W_{\gamma} \right)^{1/2}T^{-1/2}.\#(S7) \end{aligned}$$

Accordingly, the fitted slope m satisfies *m* = −*W*_γ_^1/2^ (*i.e.,* *W*_γ_ = *m*^2^). Uncertainties in *W*_γ_ were taken as the fitting error of the slope obtained from the linear regression of ln*σ*_E0_ vs *T*^−1/2^.

Figure S8. ln*σ*_E0_–*T*^−1/2^ analysis for extracting the transport-barrier parameter *W_γ_*_._ ln*σ*_E0_ as a function of *T*^–1/2^ for doped P3HT films. The *σ*_E0_(*T*) was extracted at each temperature from the *α*(*T*) and *σ*(*T*) using the K–S framework (*s* = 1) by solving Equation S3 for *η*(*T*) and using the measured *α*(*T*). Dashed lines indicate linear fits used to determine the transport-barrier parameter *W*_γ_ from the slope of ln*σ*_E0_ vs *T*^−1/2^.

Section S10. Time-dependent stability of Seebeck coefficient and power factor (RT, N_2_)

Figure S9 shows the time-dependent evolution of the Seebeck coefficient *α* and power factor *PF* for doped P3HT films measured at room temperature under N_2_. For each dopant system, *α* and *PF* were measured on the same specimen used for the conductivity analysis in Figure 5a, i.e., the highest-conductivity sample. In panel (b), the open red circles additionally denote the *PF* stability of the 0.5 mM TAB–2TFSI condition, which is the highest-*PF* sample in Figure S7. Overall, TAB–2TFSI exhibits the most stable behavior, with relatively minor changes in *α* and *PF* over extended aging. In contrast, FeCl_3_-based systems and Magic Blue show larger temporal variations, typically accompanied by a gradual reduction in *PF*, suggesting a decrease in effective doping efficiency during aging. Notably, while F_4_TCNQ:BCF yields lower initial thermoelectric performance, its *α* and *PF* remain comparatively stable over time.

Figure S9. Time-dependent stability of Seebeck coefficient and power factor of doped P3HT films measured under N_2_ at room temperature. (a) Seebeck coefficient *α* and (b) power factor *PF* as a function of aging time for P3HT films doped with TAB–2TFSI, Magic Blue, FeCl_3_/Li-TFSI, FeCl_3_, and F_4_TCNQ:BCF. In panels (a) and (b), the open red circles represent the *α* and *PF* of P3HT doped with 0.5 mM TAB–2TFSI. Error bars represent the standard deviation from three independent samples.

Figure S10. Effect of ACN rinsing on the conductivity stability of TAB–2TFSI-doped P3HT films. (a) Electrical conductivity of unrinsed and ACN-rinsed TAB–2TFSI-doped P3HT films during aging at room temperature under N₂. (b) Corresponding normalized electrical conductivity. ACN rinsing slightly decreases the initial conductivity, suggesting partial removal of weakly bound dopant-related species and/or partial perturbation of the doped state. However, the normalized conductivity retention remains nearly identical for the rinsed and unrinsed films within experimental uncertainty, indicating that removable residual species are not the dominant factor governing long-term conductivity stability. Error bars represent the standard deviation from three independent samples.

Figure S11. Time-dependent UV–Vis–NIR absorption spectra of F_4_TCNQ:BCF-doped P3HT films during aging. UV–Vis–NIR absorbance spectra of P3HT films doped with F_4_TCNQ:BCF measured after 0, 10, and 25 days of N_2_ storage (brown solid, dashed, and dash-dotted lines, respectively). The pristine P3HT spectrum is shown in gray for reference. Compared with pristine, the doped films exhibit strongly suppressed neutral vibronic absorption in the visible region (≈500–650 nm), indicating pronounced neutral bleaching that persists over the aging period. Moreover, the near-IR/sub-gap absorption remains prominent and shows only modest changes with time. However, the opposite evolution of the P2 feature near 800 nm (assigned to polarons) and the lower-energy P1 contribution near 1600 nm (assigned to both polarons and bipolarons) suggests an aging-induced redistribution of polaronic spectral weight and possible conversion of bipolarons into polarons, rather than a simple loss of the total charge carrier density. This behavior is consistent with increased carrier localization, which can reduce the mobile-carrier fraction and/or charge-transport efficiency and thereby contribute to the observed conductivity decay.

Section S11. Time-dependent GIWAXS measurement
GIWAXS data processing and linecut/peak analysis followed the procedure described in Section S4. For the aging study, independent sets of doped P3HT films were prepared inside a N_2_ glovebox for each time point (0, 10, and 25 days) and stored in sealed containers under N₂ prior to measurement. GIWAXS measurements were performed outside the glovebox; samples were opened only immediately prior to measurement, limiting air exposure to the brief handling and measurement time. The time-dependent 2D GIWAXS maps are shown in Figure S12, and the corresponding line-cut profiles are summarized in Figure S13. Time-dependent GIWAXS patterns for F_4_TCNQ:BCF-doped films are provided separately in Figure S14.

Figure S12. Time-dependent evolution of 2D GIWAXS patterns for doped P3HT films. Panels are arranged such that columns correspond to aging time (0, 10, and 25 days) and rows correspond to the dopant systems. All maps are plotted in the same reciprocal-space coordinates (*q*_xy_ and *q*_z_; Å^−1^), with the color scale representing the scattering intensity. Samples were prepared and stored under N_2_ and opened only immediately prior to measurement.

Figure S13. Line-cut GIWAXS profiles highlighting aging-dependent changes in crystalline ordering for different dopant systems. 1D intensity profiles extracted from the 2D GIWAXS patterns are shown for TAB–2TFSI (red), Magic Blue (blue), FeCl_3_/Li–TFSI (orange), and FeCl_3_ (green) at 0, 10, and 25 days (solid, dashed, and dash–dotted lines, respectively). From left to right, the panels display the in-plane *q*_ip_ cut containing the (002) and (020) reflections, and out-of-plane *q*_oop_ cuts corresponding to the (100), (200), and (300) lamellar peaks. Vertical dotted lines indicate the peak positions used for comparison.

Figure S14. Time-dependent evolution of the crystalline ordering in F_4_TCNQ:BCF-doped P3HT films. 2D GIWAXS patterns of P3HT doped with F_4_TCNQ:BCF were recorded at room temperature immediately after doping (day 0) and after 10 days of storage in an N_2_ atmosphere. GIWAXS measurements were performed outside the glovebox under ambient conditions, with air exposure limited to the brief handling and measurement period. In both conditions, the patterns are dominated by diffuse scattering with no pronounced diffraction peaks, suggesting limited long-range crystalline order in the F_4_TCNQ:BCF-doped films.

Section S12. GIWAXS analysis of additional polymer systems

GIWAXS measurements were performed on pristine, TAB–2TFSI-doped and Magic Blue-doped films of PDPP-3T, PSBTBT, P(g2T-TT), and PBTTT-C_14_ to gain structural insight. All doped films were prepared inside an N_2_ glovebox and stored in sealed containers under N₂ prior to GIWAXS measurements. Samples were unsealed immediately prior to measurement, and air exposure was limited to the brief handling and measurement time. The same fitting and analysis protocol was used to extract lattice parameters (Section S4 and Figure S1). Table S5 summarizes the extracted structural metrics—lamellar spacing (*d*_lamellar_), π–π stacking distance (*d*_π–π_), and paracrystallinity (*g*_π–π_)—for pristine and doped films. For completeness, the full polymer names are as follows: PDPP-3T (Poly{2,2′-[(2,5-bis(2-hexyldecyl)-3,6-dioxo-2,3,5,6-tetrahydropyrrolo[4-c]pyrrole-1,4-diyl)dithiophene]-5,5′-diyl-alt-thiophen-2,5- diyl}), PSBTBT (Poly[(4,4-bis(2-ethylhexyl)-dithieno[3,2-b:2′,3′-d]silole)-2,6-diyl-alt-(2,1,3-benzothiadiazole)-4,7-diyl]), P(g2T-TT) (Poly[2-(3,3′-bis(2-(2-(2-methoxyethoxy)ethoxy) ethoxy)-[2,2′-bithiophen]-5-yl)thieno[3,2-b]thiophene]), and PBTTT-C_14_ (Poly[2,5-bis(3-tetradecylthiophen -2-yl)thieno[3,2-b]thiophene]).

Table S5. GIWAXS structural parameters of additional polymer systems. The dopants used in this measurement are TAB-2TFSI and Magic Blue. All the parameters were derived from Pseudo-Voigt fits of 1D linecuts after the baseline correction as in Figure S1.

| Polymers | Pristine | | | TAB-2TFSI-doped | | | Magic Blue-doped | | |
| --- | --- | --- | --- | --- | --- | --- | --- | --- | --- |
|  | *d*_(100)_ (Å) | *d*_π–π_ (Å) | *g*_π–π_  (%) | *d*_(100)_ (Å) | *d*_π–π_ (Å) | *g*_π–π_  (%) | *d*_(100)_ (Å) | *d*_π–π_ (Å) | *g*_π–π_  (%) |
| PDPP-3T | 18.2 | 3.84 | 10.7 | 19.9 | 3.65 | 3.8 | 18.4 | 3.84 | 8.7 |
| PSBTBT | 18.5 | 3.57 | 12.7 | 19.2 | 3.50 | 12.5 | 18.8 | 3.55 | 12.4 |
| P(g2T-TT) | 16.3 | 3.56 | 9.2 | 19.1 | 3.45 | 10.6 | 17.2 | 3.50 | 9.3 |
| PBTTT-C_14_ | 21.1 | 3.68 | 8.5 | 24.5 | 3.50 | 8.4 | 22.0 | 3.65 | 8.7 |

Figure S15. AFM-IR characterization of TAB–2TFSI-doped P3HT films during aging. Topography (a,b), phase (c,d), and AFM-IR maps acquired at 1350 cm⁻^1^ (TFSI⁻-related marker band; e,f) for TAB–2TFSI-doped P3HT films measured immediately after preparation (0 day, a,c,e) and after 25 days of storage in an N_2_ glovebox (25 days, b,d,f). As indicated by the color scales in (e) and (f), higher IR signals correspond to higher TFSI⁻ concentrations. The AFM-IR maps show increased spatial contrast after aging, indicating the redistribution of TFSI-related species. The white dashed lines in (d) and (f) highlight regions with higher phase shifts (amorphous domains), which spatially correlate with regions of lower TFSI⁻ concentration. This illustrates the depletion of dopants from the amorphous phases over time. The scale bars represent 300 nm.

Figure S16. Time-dependent UV–Vis–NIR absorption spectra of pristine and doped semicrystalline polymers. Absorbance spectra of (a) PDPP-3T, (b) PSBTBT, (c) P(g2T-TT), and (d) PBTTT-C_14_ films in the pristine state (gray) and after molecular doping with TAB–2TFSI (red) or Magic Blue (blue). Doped films were prepared and stored in an N_2_ glovebox; spectra were recorded under ambient conditions immediately after doping (solid lines, 0 day) and after 25 days of glovebox storage (dashed lines, 25 days). Samples were kept in sealed containers under N_2_ and opened only immediately prior to measurement, limiting air exposure to the brief measurement period.

Table S6. Time-dependent thermoelectric properties of additional polymer systems. *σ*, *α*, and *PF* are summarized for TAB–2TFSI- and Magic Blue-doped films of PDPP-3T, PSBTBT, P(g2T-TT), and PBTTT-C_14_. Measurements were performed in an N_2_ glovebox immediately after doping (0 day) and again after 25 days of glovebox storage. All values are reported as mean ± standard deviation (*n* ≥ 3).

| Polymers | TAB–2TFSI-doped | | | Magic Blue-doped | | |
| --- | --- | --- | --- | --- | --- | --- |
|  | *σ* (S cm^−1^) | *α* (μV K^−1^) | *PF* (μW m^−1^ K^−2^) | *σ* (S cm^−1^) | *α* (μV K^−1^) | *PF* (μW m^−1^ K^−2^) |
| 0 day | | | | | | |
| PDPP-3T | 60 ± 10 | 104 ± 5 | 65 ± 9 | 71 ± 9 | 69 ± 4 | 34 ± 6 |
| PSBTBT | 71 ± 12 | 49 ± 3 | 17 ± 4 | 86 ± 10 | 55 ± 4 | 26 ± 4 |
| P(g2T-TT) | 175 ± 12 | 11 ± 2 | 2.1 ± 0.6 | 32 ± 1 | 19 ± 1 | 1.2 ± 0.2 |
| PBTTT-C14 | 548 ± 13 | 24 ± 2 | 33 ± 5 | 350 ± 20 | 28 ± 3 | 27 ± 7 |
| 25 days | | | | | | |
| PDPP-3T | 26 ± 4 | 126 ± 10 | 41 ± 9 | 16 ± 2 | 57 ± 4 | 5 ± 1 |
| PSBTBT | 48 ± 2 | 8 ± 1 | 0.3 ± 0.1 | 31 ± 2 | 8 ± 1 | 0.2 ± 0.1 |
| P(g2T-TT) | 102 ± 5 | 6.9 ± 0.3 | 0.5 ± 0.1 | 5 ± 1 | 43 ± 3 | 0.9 ± 0.2 |
| PBTTT-C14 | 321 ± 9 | 21 ± 1 | 14 ± 1 | 46 ± 12 | 32 ± 2 | 5 ± 1 |

Supporting References

[1] T. Kurosawa, T. Okamoto, Y. Yamashita, S. Kumagai, S. Watanabe, J. Takeya, *Adv. Sci.* 2021, 8, 2101998.

[2] E. H. Suh, J. G. Oh, J. Jung, S. H. Noh, T. S. Lee, J. Jang, *Adv. Energy Mater.* 2020, 10, 2002521.

[3] O. Zapata-Arteaga, A. Perevedentsev, M. Prete, S. Busato, P. S. Floris, J. Asatryan, R. Rurali, J. Martín, M. Campoy-Quiles, *ACS Energy Lett.* 2024, 9, 3567.

[4] V. Untilova, J. Hynynen, A. I. Hofmann, D. Scheunemann, Y. Zhang, S. Barlow, M. Kemerink, S. R. Marder, L. Biniek, C. Müller, M. Brinkmann, Macromolecules 2020, 53, 6314.

[5] J. Wang, Z. Zhao, S. Song, Q. Ma, R. Liu, *Polymers* 2018, 10, 1179.

[6] B. Dörling, I. E. Jacobs, I. Brunetti, N. J. Pataki, S. K. Yee, D. Scheunemann, K. Kang, G. Zuo, J. S. Reparaz, T. Mori, M. L. Chabinyc, C. Müller, M. Caironi, M. Kemerink, M. Campoy-Quiles, *Adv. Mater*. 2026, 38, e20430.

[7] S. D. Kang, G. J. Snyder, *Nat. Mater.* 2017, 16, 252–257.

[8] S. A. Gregory, R. Hanus, A. Atassi, J. M. Rinehart, J. P. Wooding, A. K. Menon, M. D. Losego, G. J. Snyder, S. K. Yee, *Nat. Mater.* 2021, 20, 1414–1421.
